# A High Dose of Dietary Berberine Improves Gut Wall Morphology, Despite an Expansion of *Enterobacteriaceae* and a Reduction in Beneficial Microbiota in Broiler Chickens

**DOI:** 10.1128/msystems.01239-22

**Published:** 2023-01-31

**Authors:** Tessa Dehau, Marc Cherlet, Siska Croubels, Filip van Immerseel, Evy Goossens

**Affiliations:** a Livestock Gut Health Team (LiGHT) Ghent, Department of Pathobiology, Pharmacology and Zoological Medicine, Faculty of Veterinary Medicine, Ghent University, Merelbeke, Belgium; b Laboratory of Pharmacology and Toxicology, Department of Pathobiology, Pharmacology and Zoological Medicine, Faculty of Veterinary Medicine, Ghent University, Merelbeke, Belgium; University of Connecticut

**Keywords:** berberine, gut health, gut microbiota, 16S rRNA gene sequencing

## Abstract

Phytogenic products are embraced as alternatives to antimicrobials, and some are known to mitigate intestinal inflammation and ensure optimal gut health and performance in broiler chickens. Dietary inclusion of berberine, a benzylisoquinoline alkaloid found in plants, is believed to exert gut health-promoting effects through modulation of the gut microbiota; however, there are only a few studies investigating its effects in chickens. The aim of this study was to investigate the interplay between dietary supplementation of a high concentration of berberine, the gastrointestinal microbiota, and histomorphological parameters in the gut. Berberine was shown to increase villus length and decrease crypt depth and CD3^+^ T-lymphocyte infiltration in the gut tissue of chickens at different ages. Berberine affected the diversity of the gut microbiota from the jejunum to the colon, both at a compositional and functional level, with larger effects observed in the large intestine. A high concentration of berberine enriched members of the *Enterobacteriaceae* family and depleted members of the *Ruminococcaceae*, *Lachnospiraceae*, and *Peptostreptococcaceae* families, as well as tended to reduce butyrate production in the cecum. *In vivo* results were confirmed by *in vitro* growth experiments, where increasing concentrations of berberine inhibited the growth of several butyrate-producing strains while not affecting that of *Enterobacteriaceae* strains. Positive correlations were found between berberine levels in plasma and villus length or villus-to-crypt ratio in the jejunum. Our study showed that berberine supplementation at a high concentration improves chicken gut morphology toward decreased inflammation, which is likely not mediated by the induced gut microbiota shifts.

**IMPORTANCE** Dietary additives are widely used to reduce intestinal inflammation and enteritis, a growing problem in the broiler industry. Berberine, with anti-inflammatory, antioxidant, and antimicrobial activity, would be an interesting feed additive in this regard. This study investigates for the first time the impact of berberine supplementation on the chicken gastrointestinal microbiota, as a potential mechanism to improve gut health, together with histological effects in the small intestine. This study identified a dose-effect of berberine on the gut microbiota, indicating the importance of finding an optimal dose to be used as a dietary additive.

## INTRODUCTION

Berberine is one of the main alkaloidal active constituents of many popular medicinal plants, such as Coptis chinensis. It has been used throughout history in Traditional Chinese Medicine for the treatment of gastrointestinal infections in humans, such as bacterial diarrhea, notably due to antibacterial properties. Berberine has been shown to promote epithelial barrier integrity and has antioxidant and anti-inflammatory effects (1, 2). Berberine improved colitis in rodent models (3, 4). In addition, this compound has been extensively investigated for its lipid-lowering and glucose-lowering properties in the context of metabolic diseases, both in animal models and in humans (5–8). It is poorly absorbed into the intestinal epithelium and therefore has a very low oral bioavailability, which questions a mechanism of direct action through uptake by host cells (9). After oral administration, most of the compound remains in the intestinal lumen, and there is growing evidence that berberine could exert its activities through interaction with the gut microbiota (10), although this has not been studied in detail when investigating gut health-promoting effects. Several studies reported that following oral administration, berberine increases the production of short-chain fatty acids (SCFAs) such as butyrate, a microbiota-derived metabolite that has anti-inflammatory effects in the gut, probably by increasing the relative abundance of butyrate-producing bacteria and stimulating the butyrate microbial pathway (11–13). The modulation of the gut microbiota was proposed as a molecular mechanism underlying the therapeutic efficacy of berberine in pathological conditions, since the efficacy was reduced in animals in which intestinal bacteria communities have been suppressed by antibiotics (11). Conversely, the gut microbiota, expressing various enzymes, can also in turn transform berberine into bioactive metabolites, which are believed to participate in the pharmacological effects of berberine (14). More particularly, berberine is converted into dihydroberberine in the lumen via a reduction reaction mediated through bacterial nitroreductases, which then reverts to berberine immediately after it enters intestinal wall tissues (15). In addition to present anti-inflammatory effects *in vivo* (16), dihydroberberine production results in increased berberine bioavailability and therefore increases pharmacological efficacy (7). Other berberine-derived metabolites have been identified in feces and plasma in humans and different animal models after oral administration of berberine, principally berberrubine, thalifendine, demethyleneberberine, and jatrorrhizine, but whether their origin is mainly from microbial or host (gut, liver) metabolism is not yet fully clear (17–20).

Gut health is of particular interest for production animals, such as broiler chickens, as a properly functioning intestinal tract is essential for the efficient conversion of feed into body mass. More particularly, the gastrointestinal microbiota plays a vital role in supporting the normal development of the gut, boosting immune responses, and protecting from intestinal pathogens, as well as a role in the digestion and utilization of nutrients (21). A dysbiotic microbiota composition is often associated with a compromised intestinal epithelial barrier and intestinal inflammation (22). In production animals, antimicrobials have been used for a long time to modulate the gut microbiota and support intestinal health. The ban on antimicrobial growth promoters in many countries worldwide, and the negative perception of the use of therapeutic antimicrobials, have encouraged the poultry industry to find alternative strategies. Most of these alternatives are feed additives among which phytochemicals have shown beneficial effects by enhancing host defense against microbial infections (23, 24). Berberine in feed has already been used in several poultry trials, up to a dose of 2,000 mg/kg feed (25–27). Berberine in drinking water (1 g/L) or via oral gavage (100 to 1,500 g/kg body weight) could suppress the reproduction of the major poultry parasite Eimeria tenella and reduce the production of oocysts as well as protect against Clostridium perfringens-induced necrotic enteritis *in vivo* (26, 28–30). Also, immunomodulatory effects of berberine (15 to 100 mg/kg body weight) in broiler chickens have been shown in multiple studies (31–33).

To date, to the best of our knowledge, no study was reported in which berberine-mediated effects on both the gut microbiota and intestinal health parameters in broilers were studied. Therefore, we investigated berberine-induced gut microbiota compositional and functional changes and the associated impact on gut morphology. Moreover, although there are a large number of studies, carried out mainly in rodent models, studying the influence of berberine on the gut microbiome (7, 13, 34–36), the evidence on the modulation of intestinal microbiota by berberine shows a considerable variability to the extent of changes both in a quantitative and qualitative manner (10), notably due to the different doses of berberine and the different models being used. For that reason, our berberine study was performed in healthy animals, so that the observed effects on the microbial populations are independent of a challenge-induced dysbiosis.

## RESULTS

### Berberine positively influences gut morphology.

To assess the effect of berberine on chicken intestinal health, we measured villus height, and crypt depth and calculated villus-to-crypt ratios in the duodenum and the jejunum, collected from chickens fed either a normal diet (control) or a 1 g berberine/kg-supplemented diet (BBR), 12- or 21-days after hatching. On day 12, dietary supplementation with berberine increased the villus height and tended to decrease the crypt depth in the duodenum, resulting in an increased villus-to-crypt ratio. This effect did not extend to the jejunum (Table 1). The duodenal crypt depth was also decreased on day 21, leading to an increase in the villus-to-crypt ratio, although not significant. The same observations were made in the jejunum on day 21: crypt depth in the BBR group was lower than in the control group, and the villus-to-crypt ratio was higher (Table 1).

**TABLE 1:** Intestinal morphology and CD3^+^ T-cell abundance in the duodenum and jejunum of chickens that received a normal diet or a diet supplemented with 1 g berberine/kg feed for 12 or 21 days posthatch^*a*^

Histologicalparameter	Day 12			Day 21		
	Control	BBR	*P* value	Control	BBR	*P* value
Duodenum						
Villus height (μm)	1,416 ± 150	1,550 ± 90	**0.0479**	1,757 ± 111	1,806 ± 183	0.6100
Crypt depth (μm)	146 ± 25	122 ± 15	0.0959	165 ± 17	126 ± 24	**0.0462**
Villus-to-crypt ratio	10.13 ± 1.64	12.98 ± 1.50	**0.0131**	10.93 ± 0.92	15.18 ± 3.12	0.0684
CD3 (area %)	6.84 ± 0.75	5.20 ± 0.87	**0.0212**	7.21 ± 1.31	6.01 ± 1.36	0.1340
Jejunum						
Villus height (μm)	598 ± 147	583 ± 107	0.3800	801 ± 144	783 ± 51	0.6450
Crypt depth (μm)	99 ± 28	73 ± 12	0.9810	119 ± 24	84 ± 12	**0.0328**
Villus-to-crypt ratio	6.40 ± 1.01	8.49 ± 1.35	0.1020	6.91 ± 1.10	9.68 ± 1.30	**0.0205**

a Data are expressed as the mean ± standard deviation (*n* = 12). Villus height and crypt depth results are based on at least 9 measurements per histological section. CD3 analysis was based on 4 microscopic fields of view per section. Bold values represent significant *P* values (*P* < 0.05). Control, normal diet; BBR, 1 g berberine/kg-supplemented diet.

The amount of CD3^+^ T-cells was determined in the duodenal tissue as a marker for intestinal inflammation. On day 12, the amount of CD3^+^-positive cells in the duodenal tissue from birds fed the berberine-supplemented diet was significantly decreased compared to the control birds, while there was no difference between groups on day 21 (Table 1).

### Berberine is detected in plasma and cecal samples after oral supplementation of berberine.

To further investigate its systemic effect and its local action in the gut, berberine was quantified using a newly developed and validated ultraperformance liquid chromatography-tandem mass spectrometry (UPLC-MS/MS) method in plasma and cecal content samples. Chickens had constant access to normal (control group) or berberine-supplemented feed (1 g/kg feed, BBR group). Negligible levels of berberine were detected in the control group (close or below limit of quantification; Text S1 in the supplemental material), showing that berberine is not produced *de novo* by the chicken or by the microbiota, and as such, values detected in the BBR group are the result only of oral supplementation and subsequent metabolization in the gut and the liver. In the supplemented group, berberine levels in plasma were similar between the two ages. However, in the cecal contents, berberine was detected at a significantly lower level on day 21 than on day 12 (Table 2).

**TABLE 2:** Concentrations of berberine in plasma and cecal content of chickens that received a normal diet or a diet supplemented with 1 g berberine/kg feed for 12 or 21 days posthatch^*a*^

	Control group			BBR group		
Berberine quantification	Day 12	Day 21	*P* value	Day 12	Day 21	*P* value
BBR in plasma (ng/mL)	1.72 ± 1.65	0.98 ± 1.08	0.3480	52.95 ± 59.23	50.93 ± 38.63	0.3390
BBR in cecal content (μg/g)	0.05 ± 0.01	0.04 ± 0.01	0.3917	168.10 ± 68.33	51.82 ± 31.49	**<0.0001**

a Data are expressed as the mean ± standard deviation. Normal diet (Control group), *n* = 6; 1 g berberine/kg-supplemented diet (BBR group), *n* = 12. Bold value represents significant *P* values (*P* < 0.05).

10.1128/msystems.01239-22.1 TEXT S1 Validation results of berberine in plasma and cecal contents by UPLC-MS/MS. The analytical method was evaluated in terms of the validation criteria described in the CMVP (EMEA/CVMP/VICH/463202/2009) and in the CHMP (EMEA/CHMP/EWP/192217/20) guidelines. Download Text S1, DOCX file, 0.03 MB. Copyright © 2023 Dehau et al. 2023 Dehau et al. https://creativecommons.org/licenses/by/4.0/ This content is distributed under the terms of the Creative Commons Attribution 4.0 International license.

### Plasma levels of berberine positively correlated with jejunal morphological parameters in the BBR group.

**FIG 1:**
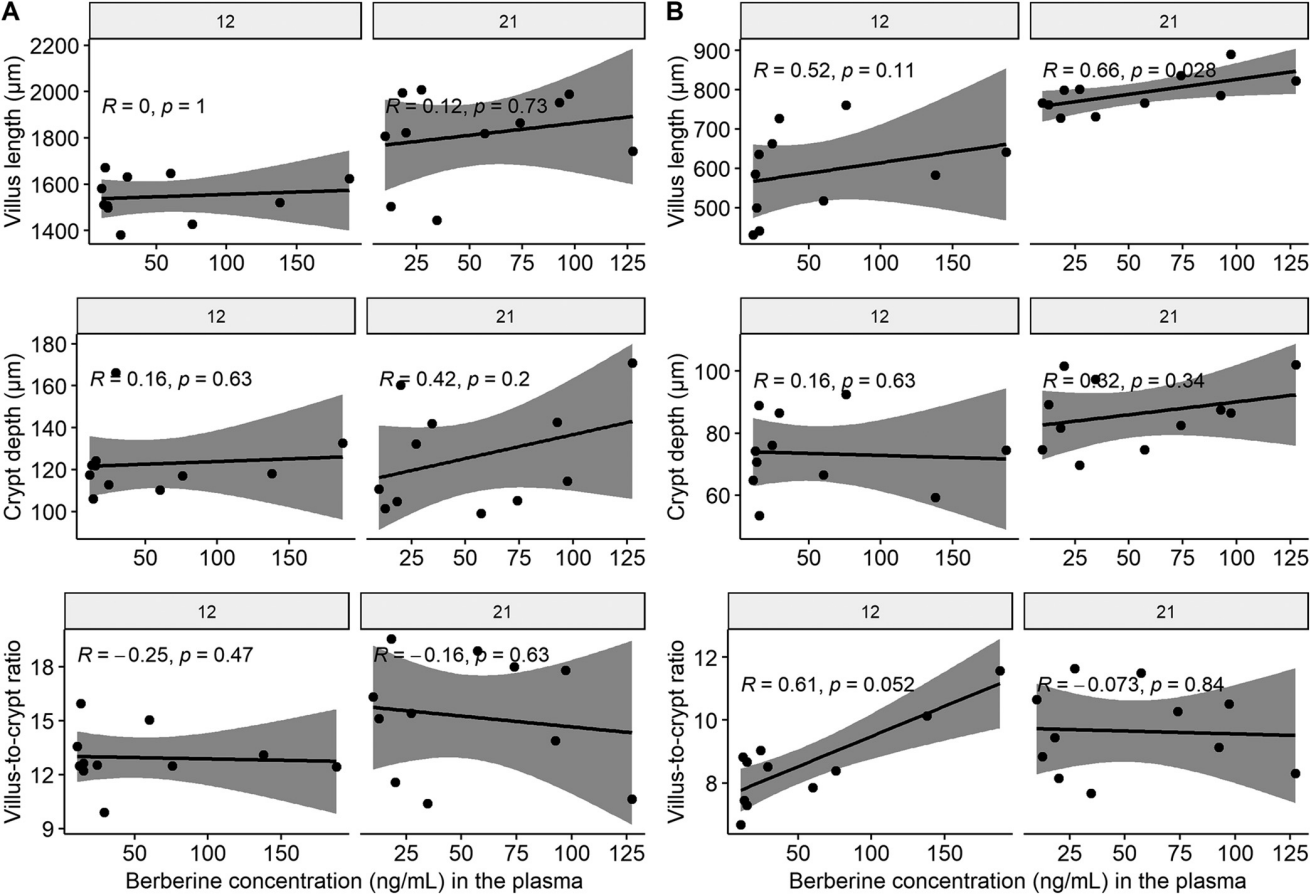
Spearman correlations between villus length, crypt depth or villus to crypt ratio in duodenum (A) or jejunum (B) and berberine concentration in plasma, in chickens that received a berberine-supplemented diet (1 g/kg feed) for 12 or 21 days posthatch. The gray shade area represents the 95% confidence interval.

To investigate whether gut wall morphological changes seen after dietary berberine administration are associated with berberine levels in the plasma or the intestinal content, a Spearman correlation analysis between berberine concentrations and either duodenal or jejunal histological parameters was carried out for chickens from the BBR group. No significant correlations were found between berberine concentrations in the cecum and the small intestinal morphology (data not shown). No significant correlations were found between plasma berberine concentrations and duodenal morphological parameters (Fig. 1A). Figure 1B shows that levels of berberine in plasma tend to have a positive correlation with the villus-to-crypt ratio (*R* = 0.61, *P* = 0.052) in the jejunum on day 12 and with the villus length (day 12: *R* = 0.52, *P* = 0.11; day 21: *R* = 0.66, *P* = 0.028) in the jejunum on both days.

### Berberine influences microbial diversity in an age-dependent manner.

**FIG 2:**
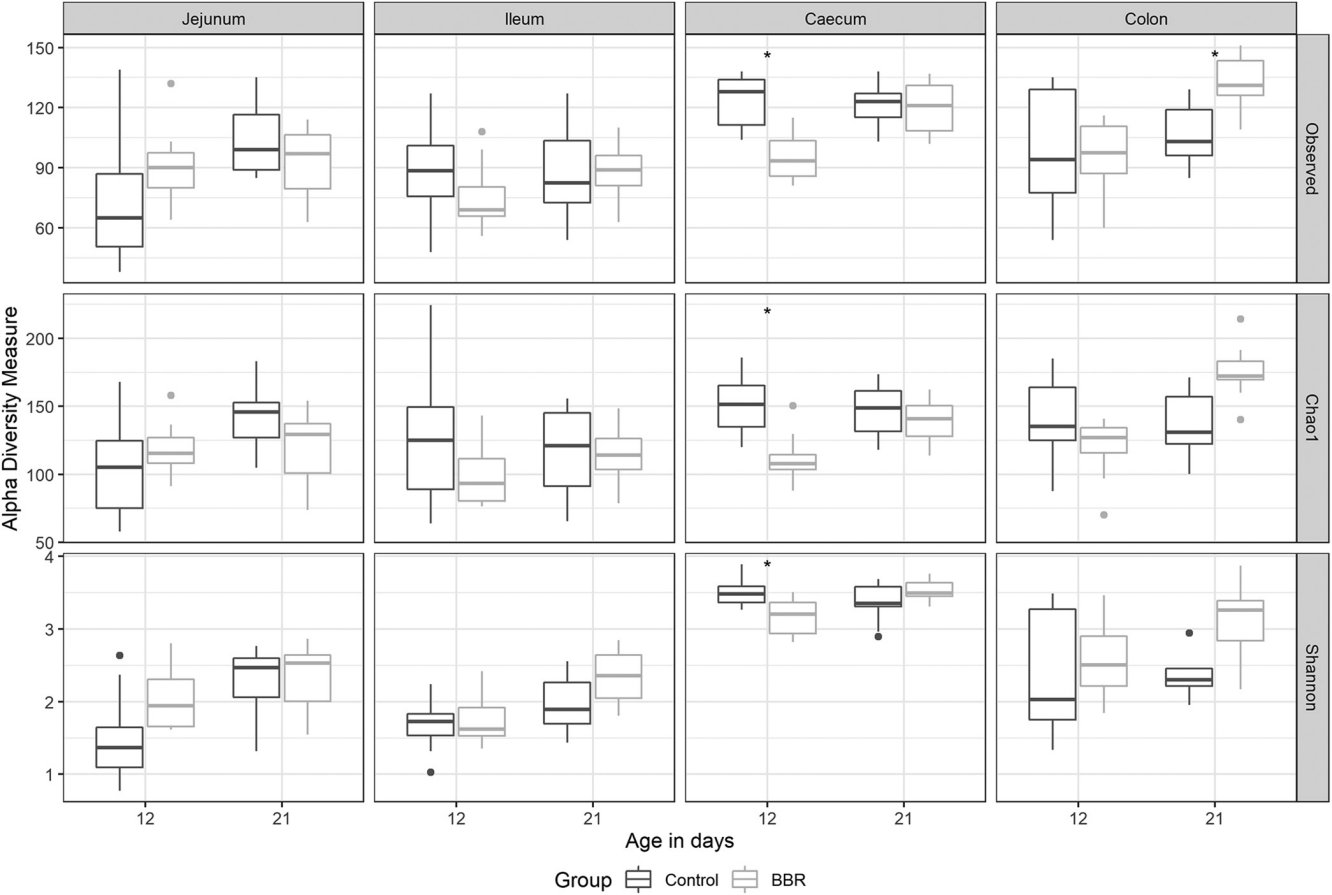
Alpha diversity of the microbial community in different intestinal segments from chickens that received a normal diet (Control) or a diet supplemented with 1 g berberine/kg feed (BBR) for 12 or 21 days posthatch. Observed, observed OTUs; Chao1, estimated species richness; Shannon, estimated species diversity. *, *P* ≤ 0.05. The Shannon index tended to increase in the jejunum in the BBR group on day 12 (*P* = 0.0722). The observed number of OTUs (*P* = 0.0114), Chao1 estimator (*P* = 0.0287), and the Shannon index (*P* = 0.0425) were significantly reduced by berberine in the cecum on day 12. The observed number of OTUs was significantly increased (*P* = 0.0297), as well as the Chao1 estimator (*P* = 0.0914) and the Shannon index (*P* = 0.0780) tended to be increased in the colon on day 21 in the BBR group.

To investigate the effect of berberine on the microbial composition, the microbiota from the jejunum, ileum, cecum, and colon of broilers from both groups was analyzed by 16S rRNA gene sequencing. Alpha diversity, including the observed number of operational taxonomic units (OTUs), the estimated OTU richness (Chao1), and the estimated community diversity (Shannon index), was measured to detect, for each intestinal segment (jejunum, ileum, cecum, and colon) and day (12 or 21), the richness and diversity of microbial communities within the samples of each group. At 12 days posthatch, the supplementation of berberine to the feed tended to increase the alpha diversity in the jejunum (Shannon index, *P* = 0.0722). This effect did not extend to the ileum whereas it was opposite in the cecum, where both the richness (observed number of OTUs, *P* = 0.0114; Chao1 estimator, *P* = 0.0287) and the diversity (Shannon index, *P* = 0.0425) were significantly decreased (Fig. 2). At 21 days posthatch, berberine only changed and increased the alpha diversity in the colon (observed number of OTUs, *P* = 0.0297; Chao1 estimator, *P* = 0.0914, Shannon index, *P* = 0.0780) (Fig. 2).

**FIG 3:**
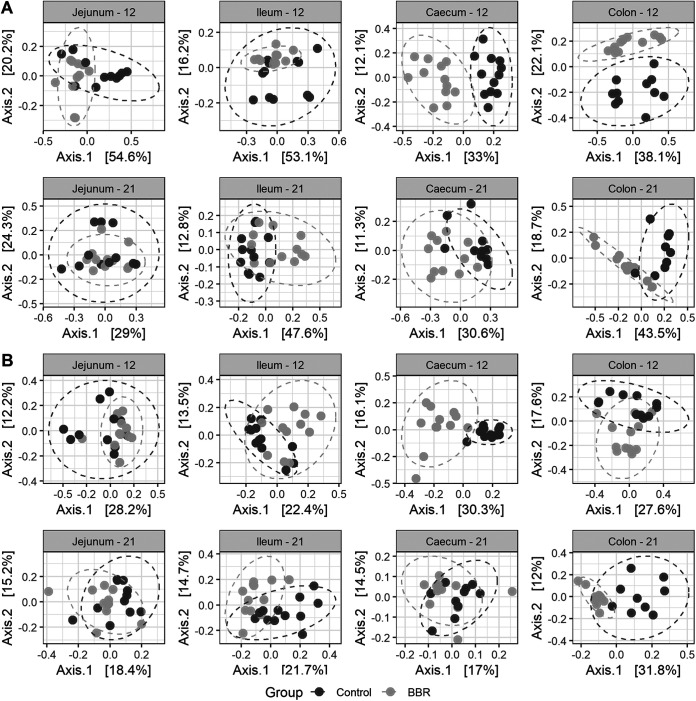
Principal coordinate analysis (PCoA) plot of beta diversity of the chicken microbiota in different intestinal segments, at days 12 and 21 of age, between the control group and the BBR group that received a diet supplemented with 1 g berberine/kg feed. Between-sample dissimilarities were measured by Bray-Curtis distance (A) or unweighted UniFrac distance (B), for each intestinal segment and for each day. Each point represents a single chicken microbiome. Significant separations of microbial communities were revealed, for all age-segment conditions, except for the jejunum on day 12 (*P* = 0.066), using PERMANOVA (Table 3).

Bray-Curtis and unweighted UniFrac dissimilarities, two classical and complementary distance metrics, were used to investigate beta diversity between the microbiota from chickens fed the control diet or the diet supplemented with berberine. Bray-Curtis accounts for relative abundances of taxa while unweighted UniFrac uses a presence/absence metric, which gives information about rare species that are possibly omitted by abundance-based methods and incorporates phylogenetic information, which is thought to improve microbial diversity estimation. This estimation was performed for every intestinal segment, first including data of both ages. Permutational analysis of variance (PERMANOVA) was done on the distance matrices and uncovered a significant interaction term between feed-additive intervention and age in all intestinal regions, showing that the effect of berberine differs depending on the age of the chicken (Table S1). Distance matrices were further calculated for every intestinal segment and age, and PERMANOVA analysis highlighted a significant shift of the bacterial taxa composition structure induced by berberine supplementation across the whole chicken intestinal tract (Table 3). This was illustrated by principal coordinate analysis (PCoA) plots, where the samples of the BBR group clustered together and clearly separated from the samples of the control group, suggesting that the microbial composition of broilers in the BBR group differs from that of the control group (Fig. 3). This suggests that the in-feed supplementation of berberine has a huge effect on the microbial community structure in both small and large intestine of chickens.

**TABLE 3:** PERMANOVA analysis of the effect of 1 g berberine/kg feed supplementation on chicken intestinal microbiota dissimilarities based on Bray-Curtis and unweighted UniFrac^*a*^

Microbiota	Bray-Curtis		Unweighted UniFrac	
	*R* ^2^	*P* value	*R* ^2^	*P* value
Day 12				
Jejunum	21.9%	**0.003**	11.5%	**0.004**
Ileum	12.4%	**0.013**	12.0%	**0.001**
Cecum	28.8%	**0.001**	25.2%	**0.001**
Colon	20.1%	**0.001**	13.7%	**0.001**
Day 21				
Jejunum	6.4%	0.066	7.2%	**0.033**
Ileum	21.2%	**0.001**	13.3%	**0.001**
Cecum	16.8%	**0.002**	10.7%	**0.001**
Colon	29.5%	**0.002**	25.4%	**0.001**

a *P* values were calculated on 999 possible permutations. Bold values represent significant *P* values (*P* < 0.05). *R*^2^ represents the percentage of variation accountable for the supplementation of berberine in the feed.

10.1128/msystems.01239-22.2 TABLE S1 PERMANOVA analysis of the effect of age and 1 g berberine/kg feed supplementation on chicken intestinal microbiota dissimilarities based on Bray-Curtis and unweighted UniFrac. Download Table S1, DOCX file, 0.01 MB. Copyright © 2023 Dehau et al. 2023 Dehau et al. https://creativecommons.org/licenses/by/4.0/ This content is distributed under the terms of the Creative Commons Attribution 4.0 International license.

### Berberine induces important shifts in the microbiota composition at all taxa levels across the intestinal tract.

**FIG 4:**
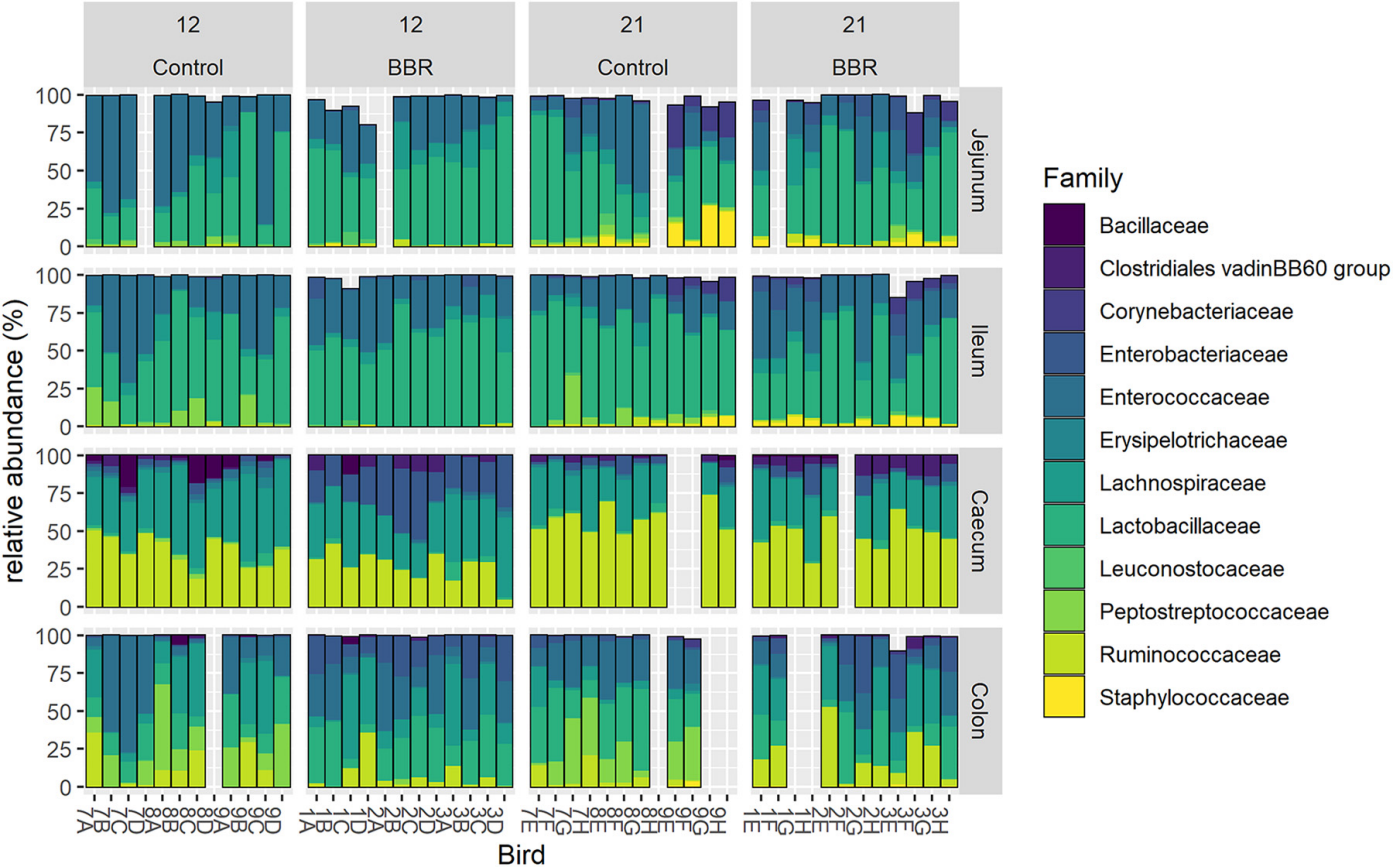
Relative abundance (%) of the 12 most abundant families in the jejunum, ileum, cecum, or colon from chickens that received a normal diet (Control) or a diet supplemented with 1 g berberine/kg feed (BBR) for 12 or 21 days posthatch. Each bar represents an individual chicken microbiome, identified on the *x* axis by a label composed by a number, representing the pen, (Control: 7, 8, 9; BBR: 1, 2, 3) and a letter, representing the age (day 12: A, B, C, D; day 21: E, F, G, H). For 13 samples, the sampling or the 16S gene sequencing failed, resulting in empty bars on the graph.

To further investigate the berberine-induced changes in microbiota composition, we studied the differences in the abundance of taxa at the phylum (Table 4), family (Table 5 and 6; Fig. 4), and genus level (Table 7 and 8), in different intestinal segments. On day 12, large berberine-mediated shifts were observed at the phylum level in all intestinal segments. The relative abundance of the *Proteobacteria* phylum was consistently increased in the ileum, cecum, and colon of birds fed a berberine-supplemented diet compared to birds receiving the control diet, while the relative abundance of the *Firmicutes* phylum was reduced in the jejunum. On day 21, there was no effect of berberine supplementation at the phylum level (Table 4).

**TABLE 4:** Relative abundances at phylum level and differentially abundant phyla between microbiota derived from chickens fed a control or 1 g/kg feed berberine-supplemented diet for 12 or 21 days posthatch^*a*^

Phylum	Day 12				Day 21			
	Mean relative abundance (%)		Log_2_ fold change	Adjusted *P* value	Mean relative abundance (%)		Log_2_ fold change	Adjusted *P* value
	Control	BBR			Control	BBR		
Jejunum								
*Actinobacteria*	0.127	0.283	0.06	0.950	7.431	5.304	−1.20	0.837
*Firmicutes*	99.332	97.389	−1.73	**0.012**	88.828	86.774	1.26	0.837
*Proteobacteria*	0.540	2.328	−0.06	0.950	3.741	7.922	0.55	0.837
Ileum								
*Actinobacteria*	0.279	0.307	0.19	0.723	3.898	3.762	−0.15	0.941
*Firmicutes*	99.287	96.897	−0.20	0.723	94.847	90.242	0.14	0.941
*Proteobacteria*	0.434	2.796	2.08	**<0.001**	1.255	5.995	1.77	0.147
Cecum								
*Actinobacteria*	0.052	0.057	−0.15	0.895	0.046	0.031	−0.10	0.826
*Firmicutes*	97.019	72.561	0.14	0.895	95.331	93.153	0.10	0.826
*Proteobacteria*	2.929	27.381	4.02	**0.002**	4.623	6.816	0.68	0.239
Colon								
*Actinobacteria*	0.036	0.203	0.70	0.391	0.938	0.514	−0.12	0.957
*Firmicutes*	99.074	82.184	−0.76	0.275	96.009	85.977	0.12	0.905
*Proteobacteria*	0.890	17.614	3.83	**0.001**	3.053	13.509	2.28	0.147

a The mean relative abundance and the log_2_ fold change (BBR/Control) of the LinDA normalized abundance of each phylum are shown. Bold values indicate statistical significance (*P* value < 0.05). Control, normal diet; BBR, 1 g berberine/kg-supplemented diet.

**TABLE 5:** Differentially abundant families in the jejunal, ileal, cecal, and colonic chicken microbiota between the Control group and the BBR group that received a diet supplemented with 1 g berberine/kg feed for 12 days posthatch^*a*^

Phylum	Class	Order	Family	Mean relative abundance (%)		Log_2_ fold change	Adjusted *P* value
				Control	BBR		
Jejunum							
*Firmicutes*	*Bacilli*	*Lactobacillales*	*Enterococcaceae*	49.803	23.909	−1.88	0.043
Ileum							<0.001
*Proteobacteria*	*Gammaproteobacteria*	*Enterobacteriales*	*Enterobacteriaceae*	0.212	2.460	3.73	0.001
*Firmicutes*	*Clostridia*	*Clostridiales*	*Peptostreptococcaceae*	7.971	0.024	−6.93	<0.001
Cecum							
*Proteobacteria*	*Gammaproteobacteria*	*Enterobacteriales*	*Enterobacteriaceae*	2.902	27.357	4.09	<0.001
*Firmicutes*	*Clostridia*	*Clostridiales*	*Peptostreptococcaceae*	1.500	0.000	−9.55	<0.001
Colon							
*Proteobacteria*	*Gammaproteobacteria*	*Enterobacteriales*	*Enterobacteriaceae*	0.745	17.421	4.76	0.014
*Firmicutes*	*Clostridia*	*Clostridiales*	*Peptostreptococcaceae*	19.587	0.331	−8.66	<0.001

a The taxonomic classification, the mean relative abundance, and the log_2_ fold change (BBR/Control) of the LinDA normalized abundance of each family are shown. Only families with *P* < 0.05 and |log_2_ fold change| >1.5 were considered biologically relevant and were included.

**TABLE 6:** Differentially abundant families in the ileal, cecal, and colonic chicken microbiota between the Control group and the BBR group that received a diet supplemented with 1 g berberine/kg feed for 21 days posthatch^*a*^

Phylum	Class	Order	Family	Mean relative abundance (%)		Log_2_ fold change	Adjusted *P* value
				Control	BBR		
Ileum							
*Firmicutes*	*Clostridia*	*Clostridiales*	*Peptostreptococcaceae*	5.348	0.010	−6.34	<0.001
Cecum							
*Firmicutes*	*Clostridia*	*Clostridiales*	*Peptostreptococcaceae*	0.531	0.000	−4.50	<0.001
Colon							
*Firmicutes*	*Clostridia*	*Clostridiales*	*Clostridiales vadinBB60 group*	0.041	1.366	4.31	0.003
*Firmicutes*	*Clostridia*	*Clostridiales*	*Peptostreptococcaceae*	22.708	0.009	−9.86	<0.001

a The taxonomic classification, the mean relative abundance and the log_2_ fold change (BBR/Control) of the LinDA normalized abundance of each family are shown. Only families with *P* < 0.05 and |log2 fold change| >1.5 were considered biologically relevant and were included. No differentially abundant families (*P* < 0.05) between groups were found in the jejunum.

**TABLE 7:** Differentially abundant genera in the jejunal, ileal, cecal, and colonic chicken microbiota between the Control group and the BBR group that received a diet supplemented with 1 g berberine/kg feed for 12 days posthatch^*a*^

Phylum	Class	Order	Family	Genus	Mean relative abundance (%)		Log_2_ fold change	Adjusted *P* value
					Control	BBR		
Jejunum								
*Firmicutes*	*Clostridia*	*Clostridiales*	*Lachnospiraceae*	*Fusicatenibacter*	0.151	0.005	−4.67	<0.001
*Firmicutes*	*Clostridia*	*Clostridiales*	*Peptostreptococcaceae*	Uncultured^*b*^	1.421	0.036	−5.11	0.002
*Firmicutes*	*Clostridia*	*Clostridiales*	*Ruminococcaceae*	*Negativibacillus*	0.031	0.000	−2.76	0.002
*Firmicutes*	*Clostridia*	*Clostridiales*	*Ruminococcaceae*	Uncultured	0.036	0.270	1.78	0.019
Ileum								
*Proteobacteria*	*Gammaproteobacteria*	*Enterobacteriales*	*Enterobacteriaceae*	Escherichia- *Shigella*	0.150	2.410	3.79	0.002
*Firmicutes*	*Clostridia*	*Clostridiales*	*Lachnospiraceae*	*Shuttleworthia*	0.033	0.017	−2.75	<0.001
*Firmicutes*	*Clostridia*	*Clostridiales*	*Lachnospiraceae*	*Lachnospiraceae NK4A136 group*	0.000	0.000	−1.72	0.002
*Firmicutes*	*Clostridia*	*Clostridiales*	*Peptostreptococcaceae*	Uncultured^*b*^	7.341	0.005	−8.66	<0.001
*Firmicutes*	*Clostridia*	*Clostridiales*	*Peptostreptococcaceae*	*Romboutsia*	0.487	0.000	−6.78	<0.001
*Firmicutes*	*Clostridia*	*Clostridiales*	*Peptostreptococcaceae*	Family_*Peptostreptococcaceae*	0.167	0.000	−4.68	<0.001
*Firmicutes*	*Clostridia*	*Clostridiales*	*Ruminococcaceae*	*Negativibacillus*	0.017	0.000	−2.46	<0.001
Cecum								
*Proteobacteria*	*Gammaproteobacteria*	*Enterobacteriales*	*Enterobacteriaceae*	Klebsiella	0.157	10.586	7.85	0.016
*Proteobacteria*	*Gammaproteobacteria*	*Enterobacteriales*	*Enterobacteriaceae*	Salmonella	0.074	0.205	4.03	0.031
*Proteobacteria*	*Gammaproteobacteria*	*Enterobacteriales*	*Enterobacteriaceae*	Family_*Enterobacteriaceae*	0.002	0.236	6.91	0.014
*Proteobacteria*	*Gammaproteobacteria*	*Enterobacteriales*	*Enterobacteriaceae*	Escherichia *-Shigella*	2.517	14.188	4.00	<0.001
*Proteobacteria*	*Gammaproteobacteria*	*Enterobacteriales*	*Enterobacteriaceae*	Proteus	0.105	1.919	7.55	0.026
*Firmicutes*	*Clostridia*	*Clostridiales*	*Lachnospiraceae*	*Shuttleworthia*	0.610	0.000	−6.44	0.031
*Firmicutes*	*Clostridia*	*Clostridiales*	*Lachnospiraceae*	*Anaerostipes*	0.619	0.010	−7.89	0.015
*Firmicutes*	*Clostridia*	*Enterobacteriales*	*Lachnospiraceae*	*[Eubacterium] hallii group*	0.860	0.064	−6.42	0.001
*Firmicutes*	*Clostridia*	*Clostridiales*	*Lachnospiraceae*	*Lachnospiraceae UCG−010*	0.193	0.000	−5.61	<0.001
*Firmicutes*	*Clostridia*	*Clostridiales*	*Lachnospiraceae*	*Lachnoclostridium*	0.867	6.364	3.91	<0.001
*Firmicutes*	*Clostridia*	*Clostridiales*	*Peptostreptococcaceae*	Uncultured^*b*^	1.419	0.000	−9.25	0.001
*Firmicutes*	*Clostridia*	*Clostridiales*	*Peptostreptococcaceae*	*Romboutsia*	0.079	0.000	−5.14	0.005
*Firmicutes*	*Clostridia*	*Clostridiales*	*Ruminococcaceae*	*Faecalibacterium*	8.050	0.002	−9.30	0.023
*Firmicutes*	*Clostridia*	*Clostridiales*	*Ruminococcaceae*	*Flavonifractor*	0.843	4.538	4.87	0.001
Colon								
*Proteobacteria*	*Gammaproteobacteria*	*Enterobacteriales*	*Enterobacteriaceae*	Klebsiella	0.042	1.894	4.91	<0.001
*Proteobacteria*	*Gammaproteobacteria*	*Enterobacteriales*	*Enterobacteriaceae*	Escherichia *-Shigella*	0.658	15.184	4.80	<0.001
*Firmicutes*	*Erysipelotrichia*	*Erysipelotrichales*	*Erysipelotrichaceae*	*Merdibacter*	0.062	0.000	−2.76	0.008
*Firmicutes*	*Clostridia*	*Clostridiales*	*Lachnospiraceae*	*Shuttleworthia*	0.249	0.002	−5.10	<0.001
*Firmicutes*	*Clostridia*	*Clostridiales*	*Lachnospiraceae*	*Lachnoclostridium*	0.561	3.037	3.49	0.005
*Firmicutes*	*Clostridia*	*Clostridiales*	*Peptostreptococcaceae*	Uncultured^*b*^	18.300	0.012	−11.36	<0.001
*Firmicutes*	*Clostridia*	*Clostridiales*	*Peptostreptococcaceae*	*Romboutsia*	1.023	0.000	−9.24	<0.001
*Firmicutes*	*Clostridia*	*Clostridiales*	*Peptostreptococcaceae*	*Terrisporobacter*	0.319	0.000	−3.06	0.044
*Firmicutes*	*Clostridia*	*Clostridiales*	*Ruminococcaceae*	*Subdoligranulum*	1.079	0.002	−3.32	0.045
*Firmicutes*	*Clostridia*	*Clostridiales*	*Ruminococcaceae*	*Faecalibacterium*	3.980	0.002	−5.79	0.005
*Firmicutes*	*Clostridia*	*Clostridiales*	*Ruminococcaceae*	*Flavonifractor*	0.101	0.679	3.78	0.006

a The taxonomic classification, the mean relative abundance, and the log_2_ fold change (BBR/Control) of the LinDA normalized abundance of each genus are shown. Only genera with *P* < 0.05 and |log_2_ fold change| >1.5 were considered biologically relevant and included.

b The uncultured bacterium belongs to the OTU4409730, the sequence of which could either be identified as *Romboutsia*, *Paraclostridium*, or *Terrisporobacter* (97% ID NCBI BLAST).

**TABLE 8:** Differentially abundant genera in the cecal and colonic chicken microbiota between the Control group and the BBR group that received a diet supplemented with 1 g berberine/kg feed for 21 days posthatch^*a*^

Phylum	Class	Order	Family	Genus	Mean relative abundance (%)		Log_2_ fold change	Adjusted *P* value
					Control	BBR		
Cecum								
*Firmicutes*	*Clostridia*	*Clostridiales*	*Peptostreptococcaceae*	Uncultured	0.400	0.000	−4.33	<0.001
Colon								
*Proteobacteria*	*Gammaproteobacteria*	*Enterobacteriales*	*Enterobacteriaceae*	Enterobacter	0.010	0.009	−2.16	0.006
*Proteobacteria*	*Gammaproteobacteria*	*Enterobacteriales*	*Enterobacteriaceae*	Proteus	0.000	0.003	−1.86	<0.001
*Firmicutes*	*Bacilli*	*Lactobacillales*	*Enterococcaceae*	*Enterococcus*	26.099	16.782	−2.85	0.004
*Firmicutes*	*Bacilli*	*Lactobacillales*	*Lactobacillaceae*	*Lactobacillus*	31.929	19.949	−2.93	0.004
*Firmicutes*	*Clostridia*	*Clostridiales*	*Peptostreptococcaceae*	Uncultured	21.075	0.006	−11.60	<0.001
*Firmicutes*	*Clostridia*	*Clostridiales*	*Peptostreptococcaceae*	*Romboutsia*	0.668	0.000	−7.61	<0.001
*Firmicutes*	*Clostridia*	*Clostridiales*	*Peptostreptococcaceae*	*Ambiguous_taxa*	0.709	0.000	−6.58	<0.001
*Firmicutes*	*Clostridia*	*Clostridiales*	*Peptostreptococcaceae*	*Terrisporobacter*	0.013	0.003	−2.47	<0.001
*Firmicutes*	*Clostridia*	*Clostridiales*	*Peptostreptococcaceae*	Family*_Peptostreptococcaceae*	0.283	0.000	−6.22	<0.001

a The taxonomic classification, the mean relative abundance and the log_2_ fold change (BBR/Control) of the LinDA normalized abundance of each genus are shown. Only genera with *P* < 0.05 and |log_2_ fold change| >1.5 were considered biologically relevant and were included. No differentially abundant genera (*P* < 0.05) were found in the jejunum or the ileum.

Differentially abundant families and genera in the microbiota from birds fed a berberine-supplemented diet compared to the control diet were identified using LinDA (Table 5 to 8). Berberine induced numerous shifts in the gut microbiota at both taxa levels. Significant changes in taxa abundance were considered biologically relevant and therefore discussed when showing an absolute log_2_ fold change >1.5. Berberine supplementation led to a large decrease in several bacterial taxa. The relative abundance of the *Peptostreptococcaceae* family was consistently reduced in the ileal, cecal, and colonic microbiota at both ages when berberine was supplemented to the diet (Table 5 and 6; Fig. 4), a difference that was mainly due to an uncultured bacterium and the *Romboutsia* genus from this family (Table 7 and 8). On day 12, the *Enterococcaceae* family was reduced in the jejunum of chickens receiving the berberine-supplemented diet compared to the control (Table 5). At the genus level on day 12, the small intestine witnessed a decrease in *Negativibacillus*, a genus from the *Ruminococcaceae* family. The cecal and colonic microbiota derived from chickens of the BBR group were also depleted with genera belonging to the *Ruminococcaceae* family, including *Faecalibacterium* and *Subdoligranulum* (Table 7). The *Shuttleworthia* genus of the *Lachnospiraceae* family was decreased in the ileum, cecum, and colon on day 12. Other members of this family were decreased, including *Fusicatenibacter* in the jejunum, *Lachnospiraceae NK4A136 group* in the ileum and *Anaerostipes*, [*Eubacterium*] *hallii group*, and *Lachnospiraceae UCG-010* in the cecum (Table 7). *Merdibacter*, a genus of the *Erysipelotrichaceae* family, was also decreased in the colon on day 12. On day 21 in the colon, *Enterococcus* and *Lactobacillus*, which were one of the most abundant genera, were reduced in relative abundance by berberine, as well as lesser abundant Enterobacter and Proteus genera of the *Enterobacteriaceae* family (Table 8).

Berberine in the diet also increased the relative abundance of several taxa. The relative abundance of the *Enterobacteriaceae* family was greatly enhanced in the ileum, cecum, and colon from 12-day-old broilers of the BBR group compared to the control (Table 5; Fig. 4). This was driven by the increase of the Escherichia-Shigella genus, as well as the increase of Klebsiella in the large intestine and Salmonella specifically in the cecum (Table 7). On day 21, neither the *Enterobacteriaceae* family nor individual genera of this family were increased in the BBR group compared to the control group (Table 6 and 8). On this day in the colon, berberine also enriched the *Clostridiales vadinBB60 group* family (Table 6). Moreover, berberine enhanced some additional members of the *Lachnospiraceae* family, *Lachnoclostridium*, and of the *Ruminococcaceae* family, *Flavonifractor*, in both the cecum and colon on day 12 (Table 7).

### Berberine modifies predicted metabolic functions of the gut microbiota and tends to affect SCFA production.

**FIG 5:**
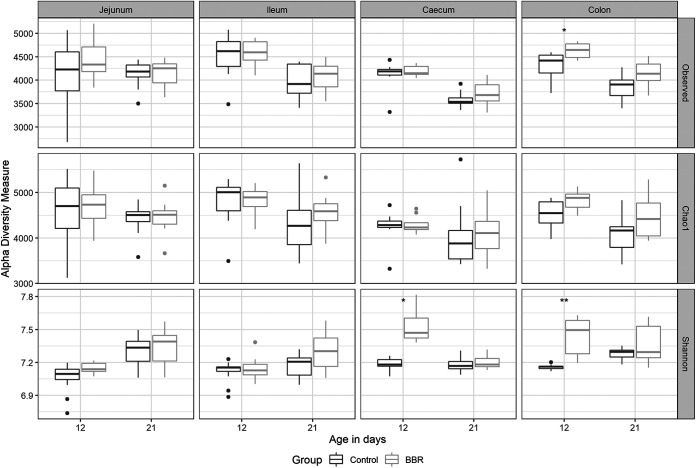
Alpha diversity of the functional microbiota in different intestinal segments, from chickens that received a normal diet (Control) or a diet supplemented with 1 g berberine/kg feed (BBR) for 12 or 21 days posthatch. Observed, observed OTUs; Chao1, estimated species richness; Shannon, estimated species diversity. *, *P* ≤ 0.05; **, *P* ≤ 0.01. On day 12, the Shannon index (*P* = 0.0223) of the cecal functional microbiota, as well as the observed number of OTUs (*P* = 0.0420) and the Shannon index (*P* = 0.0077) of the colonic functional microbiota were significantly increased in the BBR group. Berberine tended to increase the Shannon index of the jejunal functional microbiota on day 12 (*P* = 0.0926).

To determine whether the berberine-induced alterations of the microbiota might influence the microbial functions, we used PICRUST to predict *in silico* molecular functions, represented by Kyoto Encyclopedia of Genes and Genomes (KEGG) orthologues (KOs), from 16S rRNA gene profiles. Berberine did not influence the functional alpha diversity in the small intestine on both days (Fig. 5). On day 12, the functional alpha-diversity of berberine-fed chickens was increased in the cecum (Shannon index, *P* = 0.0223) and in the colon (observed KOs, *P* = 0.0420; Shannon index, *P* = 0.0077) compared to the control group. In contrast, the taxonomic alpha diversity was significantly decreased in the cecum from chickens of the BBR group, meaning that the cecal microbiome in the BBR group displayed fewer and less different taxa, which represented an equally rich, but more diverse pool of functional genes compared to the control group. On day 21, there was no significant effect of berberine on the functional repertoire in any of the intestinal segments.

**FIG 6:**
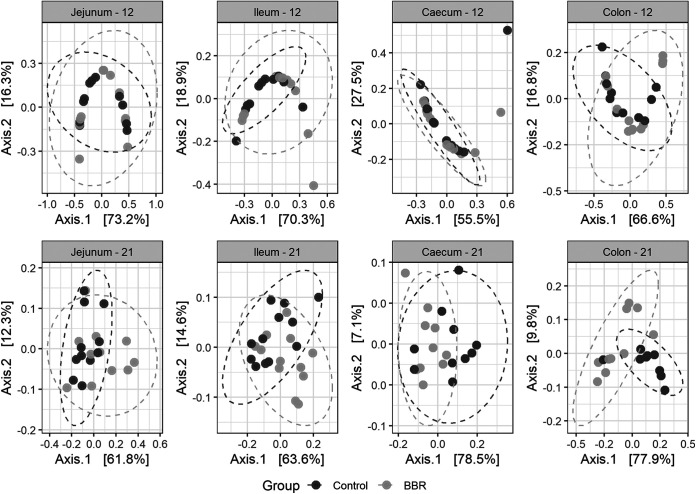
PCoA plots of beta diversity of the chicken functional microbiota in different intestinal segments, at day 12 and 21 of age, between the Control group and the BBR group that received a diet supplemented with 1 g berberine/kg feed. Between-sample dissimilarities were measured by Bray-Curtis distance for each intestinal segment and for each day. Each point represents a single chicken microbiome. Significant separation of jejunal (*P* = 0.042), cecal (*P* = 0.006), and colonic (*P* = 0.007) functional microbiota composition in 21-day-old chickens was revealed using PERMANOVA. Berberine tended to affect the functional microbiota composition in the ileum (*P* = 0.068) and in the colon (*P* = 0.085) in 12-day-old chickens (Table 9).

Bray-Curtis was used to investigate the compositional dissimilarity between the functional microbiota from chickens fed the control diet or the diet supplemented with berberine. PERMANOVA analysis revealed that at 12 days of age, berberine supplementation only tended to impact the functional community structure in the ileum and in the colon (Table 9). The PCoA plots of the KEGG orthologs abundance data indeed did not indicate a clear separation of the groups at day 12 according to the feed-additive intervention (Fig. 6). On day 21, berberine significantly changed the gene pool in the jejunum. The effect of berberine on the large intestinal functional microbiome was larger in birds at 21 days of age, where it significantly altered the functional microbiota composition in both the colon and cecum (Table 9).

**TABLE 9:** PERMANOVA analysis of the effect of 1 g berberine/kg feed supplementation on chicken gut microbiota molecular functions dissimilarities based on Bray-Curtis^*a*^

Functional microbiota	Bray-Curtis	
	*R* ^2^	*P* value
Day 12		
Jejunum	1.8%	0.648
Ileum	7.9%	0.068
Cecum	6.9%	0.110
Colon	10.7%	0.085
Day 21		
Jejunum	11.1%	**0.042**
Ileum	9.2%	0.108
Cecum	27.4%	**0.006**
Colon	31.9%	**0.007**

a *P* values were calculated on 999 possible permutations. Bold values indicate statistical significance (*P* value < 0.05). *R*^2^ represents the percentage of variation accountable for the supplementation of berberine in the feed.

**FIG 7:**
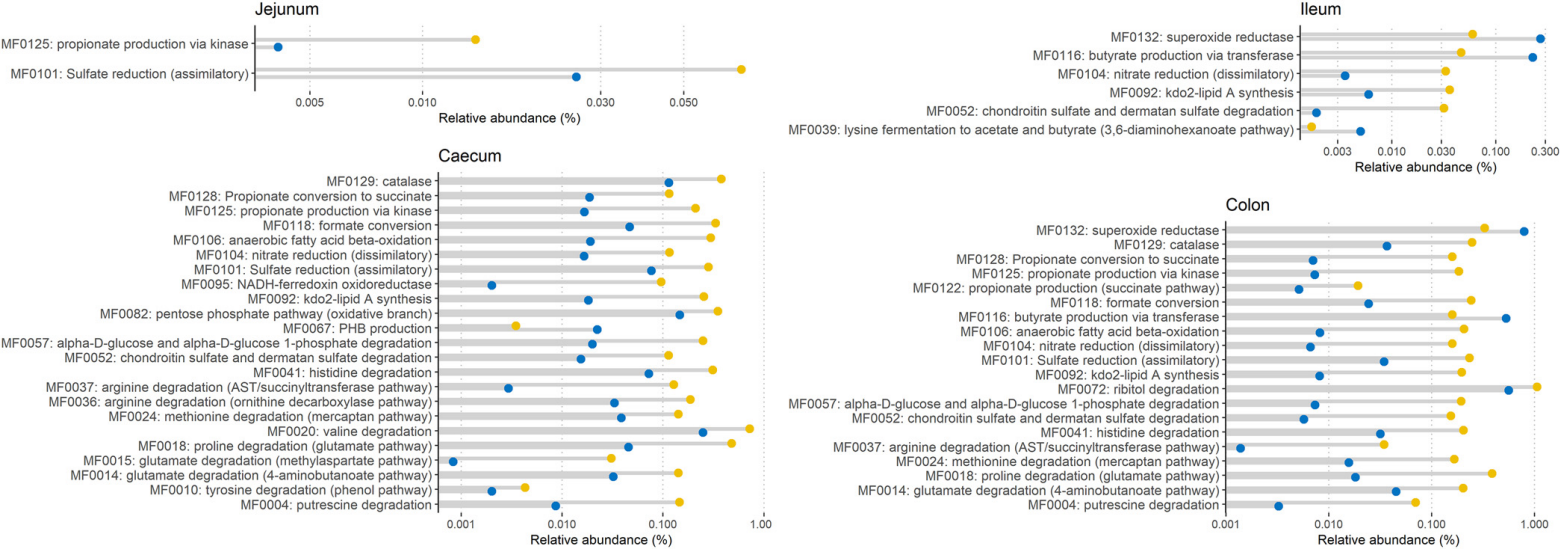
Differentially abundant metabolic modules between the Control group (blue) and the BBR group (yellow) that received a diet supplemented with 1 g berberine/kg feed for 12 days posthatch. Only metabolic modules with *P* < 0.05 and |log_2_ fold change| >1.5 were considered biologically relevant and included in the graph.

To further evaluate the berberine-induced difference in the metagenomic functional repertoire of the chicken gut, we aggregated the KO assignments into gut-specific metabolic modules (GMM). Differentially abundant functional modules between groups were observed mainly on day 12 (Fig. 7). Berberine significantly (*P* < 0.05, log_2_ fold change >1.5) enriched modules belonging to the amino-acid degradation (MF004, MF0010, MF0014, MF0015, MF0018, MF0024, MF0036, MF0037, MF0041), carbohydrate degradation (MF0052, MF0057), organic acid metabolism pathways (MF0118, MF0122, MF0125, MF0128), as well as modules related to the anaerobic respiration system (MF0095, MF0104, MF0106, MF0118) and the protection against oxidative stress (MF0129) in the cecum and the colon (Fig. 7). The *Enterobacteriaceae* family appeared as a major contributor to these overrepresented modules (Fig. S1 to S4), which is concomitant with their increased relative abundance. In the ileum, the butyrate production via transferase (MF0116) and the lysine fermentation to acetate and butyrate (MF0039) were relatively reduced. MF0116 was also reduced in the colon. Only minor effects of berberine were observed on the pool of modules on day 21 (Fig. S5).

10.1128/msystems.01239-22.3 FIG S1 Bacterial families responsible for the differentially abundant functional modules in the jejunum between the control group and the BBR group that received a diet supplemented with 1 g berberine/kg feed for 12 days. Metagenome contributions on the family level are sorted per functional module and per treatment (control, dark gray; BBR, light gray). The log_2_ of the module counts per family is shown on a blue-red scale. MF0101, sulfate reduction (assimilatory); MF0125, propionate production via kinase. Download FIG S1, TIF file, 12.1 MB. Copyright © 2023 Dehau et al. 2023 Dehau et al. https://creativecommons.org/licenses/by/4.0/ This content is distributed under the terms of the Creative Commons Attribution 4.0 International license.

10.1128/msystems.01239-22.4 FIG S2 Bacterial families responsible for the differentially abundant functional modules in the ileum between the control group and the BBR group that received a diet supplemented with 1 g berberine/kg feed for 12 days. Metagenome contributions on the family level are sorted per functional module and per treatment (control, dark gray; BBR, light gray). The log_2_ of the module counts per family is shown on a blue-red scale. MF0039, lysine fermentation to acetate and butyrate (3,6-diaminohexanoate pathway); MF0052, chondroitin sulfate and dermatan sulfate degradation; MF0092, kdo2-lipid A synthesis, MF0104: nitrate reduction (dissimilatory); MF0116, butyrate production via transferase; MF0132, superoxide reductase. Download FIG S2, TIF file, 12.1 MB. Copyright © 2023 Dehau et al. 2023 Dehau et al. https://creativecommons.org/licenses/by/4.0/ This content is distributed under the terms of the Creative Commons Attribution 4.0 International license.

10.1128/msystems.01239-22.5 FIG S3 Bacterial families responsible for the differentially abundant functional modules in the cecum between the control group and the BBR group that received a diet supplemented with 1 g berberine/kg feed for 12 days. Metagenome contributions on the family level are sorted per functional module and per treatment (control, dark gray; BBR, light gray). The log_2_ of the module counts per family is shown on a blue-red scale. MF004, putrescine degradation; MF0010, tyrosine degradation (phenol pathway); MF0014, glutamate degradation (4-aminobutanoate pathway); MF0015, glutamate degradation (methylaspartate pathway); MF0018, proline degradation (glutamate pathway); MF0020, valine degradation; MF0024, methionine degradation (mercaptan pathway); MF0036, arginine degradation (ornithine decarboxylase pathway); MF0037, arginine degradation (AST/succinyltransferase pathway); MF0041, histidine degradation; MF0052, chondroitin sulfate and dermatan sulfate degradation; MF0057, alpha-d-glucose and alpha-d-glucose 1-phosphate degradation; MF0067, PHB production; MF0082, pentose phosphate pathway (oxidative branch); MF0092, kdo2-lipid A synthesis; MF0095, NADH-ferredoxin oxidoreductase; MF101, sulfate reduction (assimilatory); MF0104, nitrate reduction (dissimilatory); MF0106, anaerobic fatty acid beta-oxidation; MF0118, formate conversion; MF0125, propionate production via kinase; MF0128, propionate conversion to succinate; MF0129, catalase. Download FIG S3, TIF file, 18.1 MB. Copyright © 2023 Dehau et al. 2023 Dehau et al. https://creativecommons.org/licenses/by/4.0/ This content is distributed under the terms of the Creative Commons Attribution 4.0 International license.

10.1128/msystems.01239-22.6 FIG S4 Bacterial families responsible for the differentially abundant functional modules in colon between the control group and the BBR group that received a diet supplemented with 1 g berberine/kg feed for 12 days. Metagenome contributions on the family level are sorted per functional module and per treatment (control, dark gray; BBR, light gray). The log_2_ of the module counts per family is shown on a blue-red scale. MF0004, putrescine degradation; MF0014, glutamate degradation (4-aminobutanoate pathway); MF0018, proline degradation (glutamate pathway); MF0024, methionine degradation (mercaptan pathway); MF0037, arginine degradation (AST/succinyltransferase pathway); MF0041, histidine degradation; MF0052, chondroitin sulfate and dermatan sulfate degradation; MF0057, alpha-d-glucose and alpha-d-glucose 1-phosphate degradation; MF0072, ribitol degradation; MF0092, kdo2-lipid A synthesis; MF0101, sulfate reduction (assimilatory); MF0104, nitrate reduction (dissimilatory); MF0106, anaerobic fatty acid beta-oxidation; MF0116, butyrate production via transferase; MF0118, formate conversion; MF0122, propionate production (succinate pathway); MF0125, propionate production via kinase; MF0128, propionate conversion to succinate; MF0129, catalase; MF0132, superoxide reductase. Download FIG S4, TIF file, 18.1 MB. Copyright © 2023 Dehau et al. 2023 Dehau et al. https://creativecommons.org/licenses/by/4.0/ This content is distributed under the terms of the Creative Commons Attribution 4.0 International license.

10.1128/msystems.01239-22.7 FIG S5 Differentially abundant metabolic modules in the colon between the Control group (blue) and the BBR group (yellow) that received a diet supplemented with 1 g berberine/kg feed for 21 days posthatch. Only metabolic modules with *P* < 0.05 and |log_2_ fold change| >1.5 were considered biologically relevant and included in the graph. No significant differences in the jejunal, ileal, or cecal microbiota functions were found between groups. Download FIG S5, TIF file, 12.1 MB. Copyright © 2023 Dehau et al. 2023 Dehau et al. https://creativecommons.org/licenses/by/4.0/ This content is distributed under the terms of the Creative Commons Attribution 4.0 International license.

To complement the analysis performed on the predicted metabolic functions, SCFAs were quantified in the cecal content from chickens of the control or BBR group (Table 10). Acetate was the major SCFA, followed by butyrate as the second most abundant SCFA. At both ages, berberine supplementation tended to decrease butyrate levels in the cecum of broilers (day 12, *P* = 0.0882; day 21, *P* = 0.0817), which was consistent with the decrease of the MF0116 module related to butyrate production (Fig. 7).

**TABLE 10:** Short-chain fatty acid concentrations in cecal digesta from chickens that received a normal diet or a diet supplemented with 1 g berberine/kg feed for 12 or 21 days posthatch^*a*^

	Day 12			Day 21		
SCFA	Control	BBR	*P* value	Control	BBR	*P* value
Acetate	50.25 ± 12.04	40.36 ± 14.13	0.2831	58.86 ± 19.54	60.87 ± 15.55	0.8638
Propionate	2.36 ± 0.79	1.51 ± 1.50	0.4116	2.40 ± 1.08	2.70 ± 1.43	0.7279
Butyrate	11.39 ± 4.05	5.96 ± 0.63	0.0882	20.94 ± 9.29	12.94 ± 2.52	0.0817
Isobutyrate	0.14 ± 0.22	0.02 ± 0.05	0.3970	0.13 ± 0.14	0.16 ± 0.32	0.8580
Valerate	0.17 ± 0.32	0.06 ± 0.15	1.0000	0.30 ± 0.18	0.13 ± 0.08	0.2215
Isovalerate	0.16 ± 0.20	0.03 ± 0.06	1.0000	0.13 ± 0.13	0.07 ± 0.12	0.4420

a Data are expressed as the mean short-chain fatty acid (SCFA) concentration (μmol/g digesta) ± standard deviation. Normal diet (Control group), *n* = 6; 1 g berberine/kg-supplemented diet (BBR group), *n* = 6.

### Berberine increases bacterial nitroreductase potential in the cecum.

**FIG 8:**
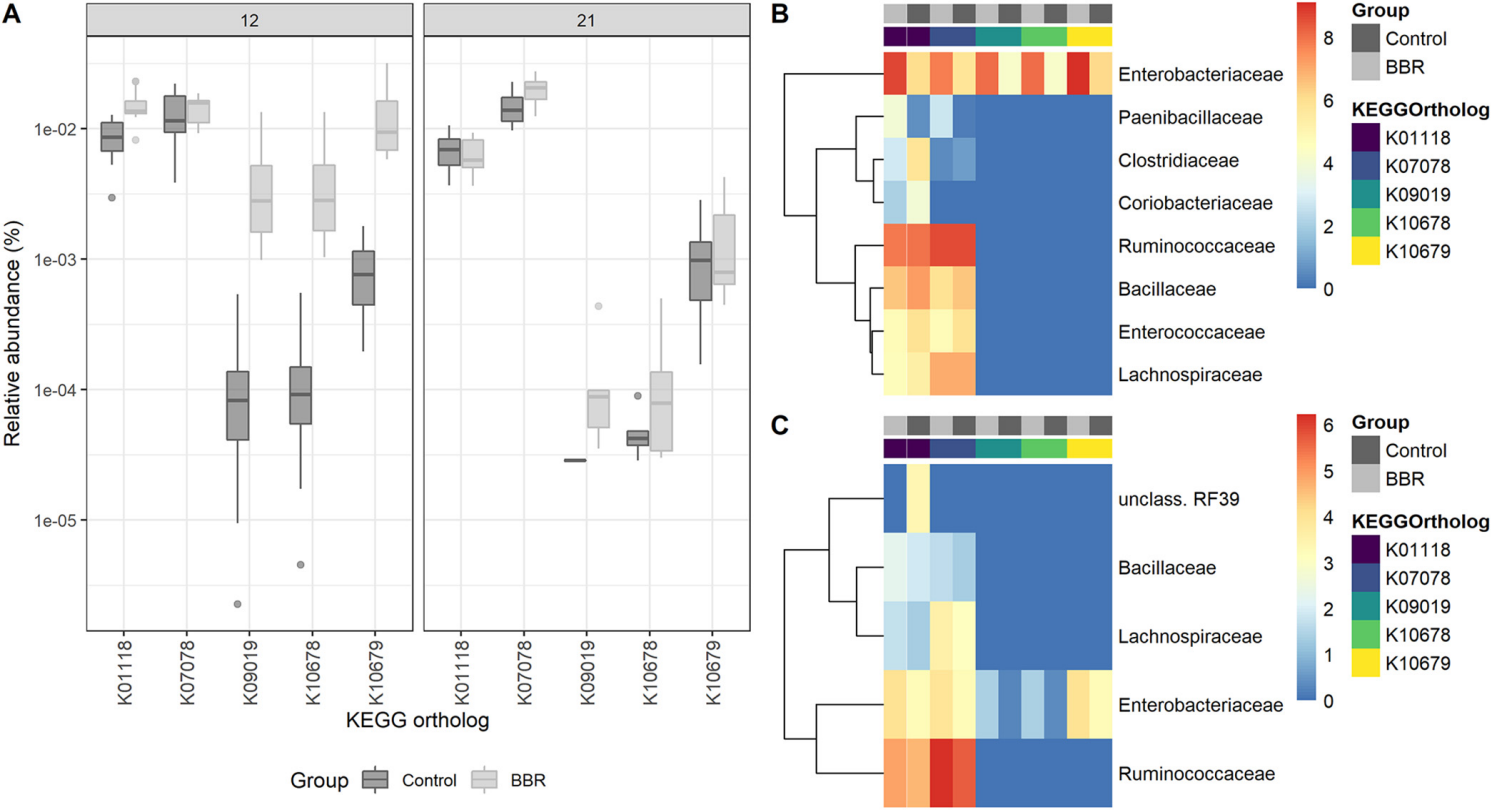
Nitroreductase activity in the cecum. (A) Relative abundance of KEGG orthologs associated with enzymes of the nitroreductase family and azoreductase in the functional microbiota in the cecum, from chickens that received a normal diet (Control) or a diet supplemented with 1 g berberine/kg feed (BBR) for 12 or 21 days posthatch. K10679 (*P* = 0.0881) and K01118 (*P* = 0.0587) tended to be increased by berberine on day 12. (B) Bacterial families responsible for the nitroreductase activity in the cecum on day 12. (C) Bacterial families responsible for the nitroreductase activity in the cecum on day 21. Metagenome contributions on the family level are sorted per KO and per treatment (control, dark gray; BBR, light gray). The log_2_ of the gene counts per family is shown on a blue-red scale. K00118, azoreductase; K07078, nitroreductase_4; K09019: nitroreductase_5; K10678, nfSB-like nitroreductase; K10679, nfSA/FRP (Nitro_FMN_reductase Conserved Protein Domain Family, NCBI).

While berberine influences gut microbial functions, berberine has also been previously shown to be converted to dihydroberberine in the gut by bacterial nitroreductases (15). To explore the presumable metabolic effect of the gut microbiota on berberine, five KOs were preselected as representative for nitroreductase activity, and their relative abundance was assessed in the cecum. Two nitroreductase-related KOs (K01118, *P* = 0.0587; K10679, *P* = 0.0881) tended to be overrepresented in the BBR group on day 12 compared to the control (Fig. 8A), for which the *Enterobacteriaceae* family was the major contributor (Fig. 8B). The nitroreductase potential was not increased in the BBR group on day 21, and this time *Ruminococcaceae* was the main contributor to the nitroreductase-related KOs, and *Enterobacteriaceae* contributed to a lesser extent (Fig. 8C).

### High concentrations of berberine exert antibacterial effect on strict anaerobes.

**FIG 9:**
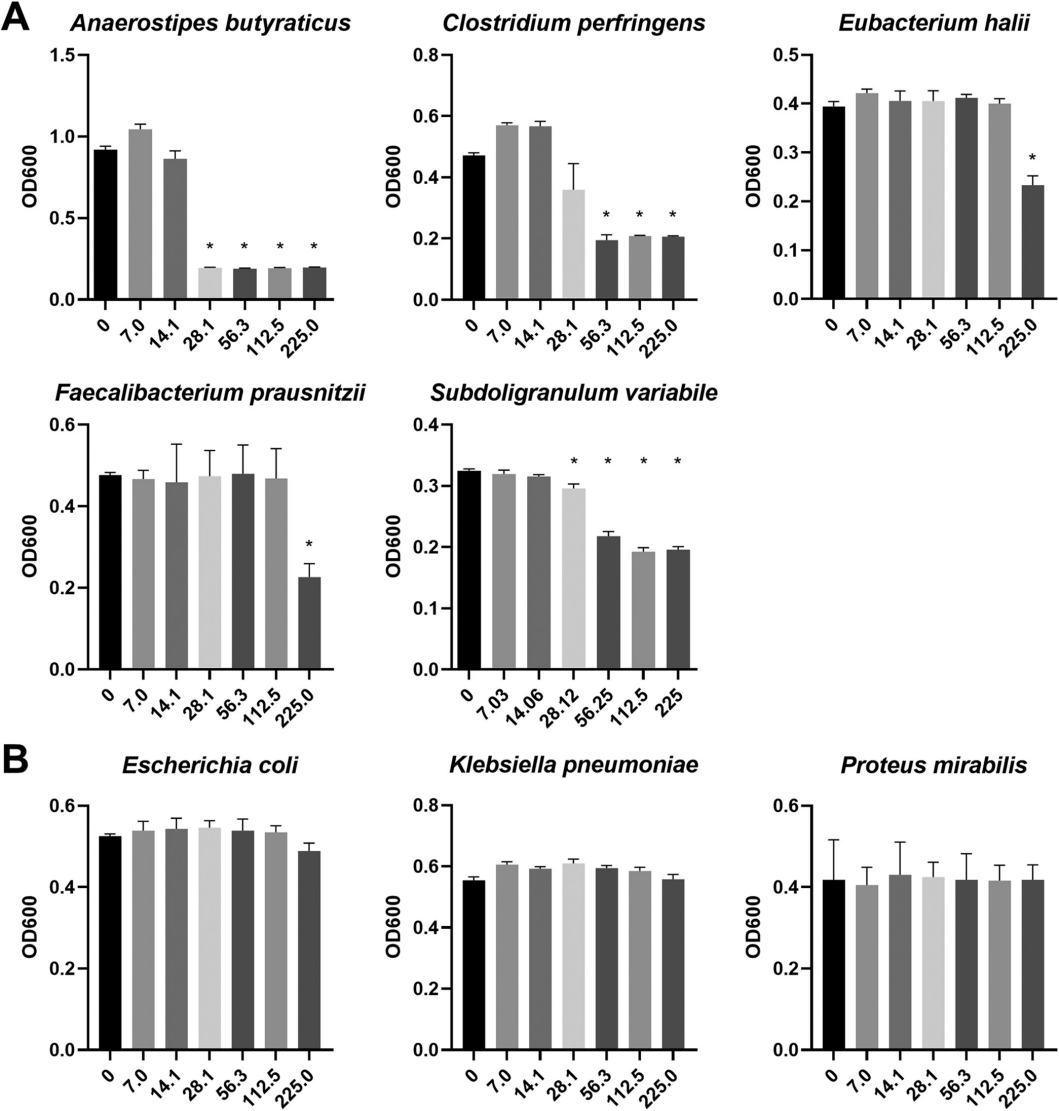
Growth of strict anaerobes (A) and facultative anaerobes (B) in the presence of increasing concentrations of berberine in M2GSC medium (μg/mL) under anaerobic conditions. The selected strains correspond to genera that were differentially abundant *in vivo* between the Control group and the BBR group. The relative abundance of genera *Anaerostipes*, *Eubacterium*, *Faecalibacterium*, and *Subdoligranulum* was reduced by a high concentration of berberine *in vivo*, whereas the relative abundance of genera Escherichia, Klebsiella, and Proteus was increased. C. perfringens was added to the collection of strains tested as berberine was previously shown to alleviate C. perfringens-induced necrotic enteritis *in vivo*. Cultures were grown for 24 h. Absorbance data at 600 nm are expressed as the mean ± standard deviation (*n* = 3). *, Significantly reduced growth compared to the nonsupplemented medium (*P* < 0.05).

To investigate whether the berberine-induced effect on the gut microbiome is linked to a direct antimicrobial effect, the effect of BBR on bacterial growth was evaluated *in vitro*. Therefore, a selection of bacterial taxa that were highly differentially abundant in the *in vivo* trial was grown in the presence of increasing concentrations of berberine, ranging from 7.1 to 225.0 μg/mL, representative of the concentrations measured in the cecal digesta from chickens fed a diet supplemented with 1 g berberine/kg feed (Fig. 9). None of the strains were affected by the two lowest concentrations of berberine tested, 7.1 and 14.1 μg/mL. Higher concentrations significantly inhibited the growth of anaerobes whereas no relevant effect was noticed on the growth of *Enterobacteriaceae* strains. Growth inhibition started at 28.1 μg/mL berberine for *A. butyraticus* and *S. variabile* and at 56.3 μg/mL for C. perfringens. Growth of *F. prausnitzii* and *E. hallii* was repressed in the presence of the highest concentration of berberine, 225 μg/mL.

## DISCUSSION

### Berberine exhibits low plasma concentration after oral administration, which can be partly explained by its metabolization in the gut.

In our study, animals were daily exposed to a relatively high dose of berberine and berberine reached a very low concentration of about 50 ng/mL in the plasma. While this concentration remained steady in the plasma between the two ages, levels detected in cecal contents were significantly lower at 21 than at 12 days of age. A study in rats showed that the absorbed fraction of berberine after administration into the duodenum was drastically less than after administration into the liver vein, suggesting that the intestinal metabolism of berberine was tremendous in rats (9). Both host and microbial enzymes can contribute to the transformation of berberine in the intestinal compartment. Recent studies found that the gut microbiota, acting as a metabolic organ, could convert berberine into different metabolites though demethylation, reduction, and demethylenation. The metabolites are usually more lipophilic and could be absorbed more efficiently in the intestine than berberine (14, 15, 37). Moreover, berberine has been shown to be a substrate of the P-glycoprotein efflux transporter, more commonly referred as multidrug resistance protein 1 (MDR1), in rats and in Caco-2 cells. MDR1 is expressed in the apical membrane of the epithelial layer of the gut wall and can actively transport certain compounds in the blood-to-lumen direction. This efflux pump may be involved in the excretion of berberine back into the intestinal lumen, leading to poor absorption (38). Its expression has been shown to be significantly higher at 4 weeks than at 2 weeks of age in broiler small intestine, whereas in our study the amount of berberine in the lumen of 3-week-old broilers was lower than younger birds. After absorption, berberine distributes widely in the tissues and mostly in the liver where it is metabolized by cytochromes P450, additional evidence to explain its low plasma exposure (39, 40). Therefore, the smaller amount of berberine in the cecum of older birds might be partly explained by a more extensive intestinal metabolism. Further research is needed to investigate the extent to which the gut environment transforms berberine as well as the repartition of berberine and its metabolites in the intestinal contents and plasma/tissues, especially since the latter are believed to contribute to berberine pharmacological effects (14).

### Supplementation of berberine to the diet induces changes in intestinal morphology and inflammation that are linked to beneficial gut health and animal performance.

Villus height, crypt depth, and villus-to-crypt ratio, measured at the level of the duodenum, jejunum, or ileum, are widely used as the standard readout for the evaluation of intestinal health in poultry studies (41). Broilers fed berberine demonstrated lower crypt depth in the duodenum across the trial, resulting in an increased villus-to-crypt ratio. Berberine also increased duodenal villus height and reduced inflammatory T-cell infiltration in the duodenal tissue in younger birds. The broiler intestines are constantly exposed to various challenges that affect intestinal barrier integrity and can trigger inflammation (e.g., coccidia, mycotoxins, bacterial toxins, among others), resulting notably in villus atrophy (42). In response, the host increases epithelial cell turnover in the intestinal mucosal crypts to permit renewal of the villi via migration of cells from the crypts to the villus tip, which can translate as deeper crypts (43). Intestinal challenge models typically result in shorter villi, deeper crypts, and more infiltration of T lymphocytes in association with performance losses (44). In the present study, the reduction of crypt size in the small intestine of broilers fed a berberine-supplemented diet, along with the improved villus height in the duodenum, may indicate that there was less epithelial cell loss at the villus tip compared to the control. Therefore, dietary berberine-supplementation might support intestinal health and ensure optimal performance by enhancing intestinal barrier integrity and controlling inflammation in the avian gut.

### Berberine given as dietary additive at high dose induces dysbiosis-like shifts in the intestinal microbiota.

Berberine supplementation resulted in a relative expansion of *Proteobacteria* (mainly family *Enterobacteriaceae*) and a depletion of *Firmicutes*, more particularly obligate anaerobes of the families *Ruminococcaceae*, *Lachnospiraceae*, and *Peptostreptococcaceae*. Initially colonized by *Enterobacteriaceae*, the hindgut of newly hatched chicks is normally progressively, during the second week of life, dominated with *Lachnospiraceae* and *Ruminococcaceae* from the phylum *Firmicutes*, and therefore, the prevalence of *Enterobacteriaceae* remains low (45, 46). On the contrary, in our study, this family accounted for 27.4% and 17.4% of the total sequences of the cecal and colonic microbiota of 12-day-old chickens that received a berberine-supplemented diet, i.e., more than a 16-fold increase compared to the control. In the cecum, this was associated with a lower diversity of bacteria carrying disparate functional genes, characteristic of an immature microbiome. A mature microbiome on the contrary includes more diverse bacterial taxa that can perform similar functional roles, representing a more specialized community (46). Expansion of *Proteobacteria* has been observed as a result of antibiotic treatment or inflammation in animals and humans and is considered a microbial signature of gut dysbiosis and epithelial dysfunction (47). During gut homeostasis, beta-oxidation of microbiota-derived butyrate causes epithelial hypoxia, which maintains anaerobiosis in the lumen of the hindgut and in turn drives a dominance of obligate anaerobic bacteria within the gut microbiota. Depletion of butyrate-producing bacteria, as we observed in our study, including *Anaerostipes*, [*Eubacterium*] *hallii group*, *Faecalibacterium*, and *Subdoligranulum*, often reduces luminal butyrate levels, although this did not reach statistical significance in our study (*P* = 0.0882), which results in a metabolic reorientation of surface colonocytes toward fermentation of glucose, thereby increasing oxygenation of the epithelium and finally driving the expansion of facultative anaerobic *Enterobacteriaceae* through aerobic respiration (48). This was supported by our *in vitro* study showing a selective antibacterial effect of berberine against the anaerobes, while it was indifferent as to the facultative anaerobes E. coli, K. pneumoniae, and P. mirabilis in the range 7 to 225 μg/mL, concentrations resembling *in vivo* conditions (168.10 ± 68.33 μg/g cecum content). Our results are consistent with a previous study that reported MIC values superior to 500 μg/mL for the latter *Enterobacteriaceae* strains (15). In addition, *in vivo* microbial functions presumably related to nitrate respiration (formate conversion, anaerobic fatty acid beta oxidation, and nitrate reduction, where formate and fatty acids are all potential electron donors for nitrate reduction) were upregulated in the BBR group, and the *Enterobacteriaceae* family was the biggest contributor to these functional modules. This suggests that the bacteria of this family respired nitrate as an alternative electron acceptor to outgrow obligate anaerobes. Research showed that butyrate suppresses the synthesis of host-derived nitrate; therefore, the trend in the reduction of butyrate in our study might have led to an increased nitrate production which finally permitted the observed bloom of *Enterobacteriaceae* (49).

### The other remarkable berberine-mediated effect of the gut microbiota was the reduction in relative abundance of the family *Peptostreptococcaceae* and the corresponding genus *Romboutsia*.

The family was present in every investigated intestinal compartment in both ages and more particularly was the major colonizer in the colon of chickens, approximating 20% of the total sequences isolated from this intestinal part but was drastically eradicated from the gut lumen after dietary berberine supplementation. Increased abundance of *Peptostreptococcaceae* has been associated with inflammatory conditions such as ulcerative colitis (50) and colorectal cancer (51). More particularly, *Romboutsia* belongs to the *Clostridium cluster XI* within the *Peptostreptococcaceae* family, which contains harmful bacteria in the large intestine, including the well-known pathogen *Clostridoides difficile*. However, the functional role of this genus in the intestinal tract is still unclear, as it was defined only recently (52). In general, in the context of berberine treatment, the family *Peptostreptococcaceae* has not been described before, but previous studies found a significantly reduced abundance of *Clostridium cluster XI* associated with berberine treatment (53). A short-term study investigating the effect of metformin, a clinically effective drug for treating diabetes, on the composition of healthy human gut microbiota highlighted a relative increase of the genus Escherichia-Shigella and a decrease of the *Peptostreptococcaceae* and four genera within it, among which the genus *Romboutsia* (54). Zhang et al. (55) previously showed that berberine showed similarity in modulating the gut microbiota with metformin. Therefore, berberine-mediated reduction of members of the *Peptrostreptococcaceae* in the gut could translate into an anti-inflammatory effect, although further research is needed to characterize the functional role of this family in the gut ecosystem.

### The improvement of broiler gut morphology following berberine supplementation seems to be independent of the microbiome.

The correlation analysis revealed that increasing berberine levels in plasma were linked to longer villi and higher villus-to-crypt ratio in the jejunum from birds of the BBR group. This suggests that direct interaction of berberine with host cells, through absorption in the gut tissue and distribution in the peripheral blood, is partly responsible for the changes in intestinal morphology observed in this study. Li et al. (56) demonstrated in *in vitro* experiments with Caco-2 monolayers that berberine can ameliorate proinflammatory cytokine-induced intestinal epithelia tight junction damage, which could explain its ability to prevent enterocyte loss. On the other hand, microbial shifts induced by berberine do not seem to be related to the observed effects on gut morphology. A dysbiosis as observed in the current work has been previously associated in broilers with villus atrophy and an increased T-lymphocyte infiltration in the gut mucosa (42), which is in contradiction with what we observed. In addition, butyrate, a key molecule to promote gut health, tended to be reduced in the present study. Besides the maintenance of anaerobiosis, butyrate is also capable of enhancing epithelial integrity and reducing inflammation via modulation of proinflammatory cytokines as well as negatively affecting the expression of virulence factors of bacterial pathogens (57, 58). Berberine-mediated gut microbiota shifts would therefore point toward a poor intestinal microarchitecture. However, we hypothesize that the production of bioactive berberine-derived metabolites by microbial metabolism could contribute to observed effects. Berberine was shown to be more extensively metabolized *in vitro* by a diarrheal intestinal microbiota, characterized by increased E. coli counts, likely due to an increase in the activity of microbial nitroreductases, enzymes recognized as involved in berberine metabolism and the production of dihydroberberine (59). The latter metabolite showed anti-inflammatory effects in a colitis model (16). More particularly, several *Enterobacteriaceae* genera have been described as efficient producers of these enzymes (7, 15, 60). In our study, berberine seemed to improve the relative abundance of several KOs related to nitroreductase activity, with *Enterobacteriaceae* genera counting as the major contributors to this activity. Although commonly associated with adverse health effects, the stimulation of the latter family by high concentrations of berberine might therefore lead to the production of berberine-derived bioactive metabolites and contribute to *in vivo* effects.

### Berberine-induced gut microbiota alterations might be dose dependent.

The influence of berberine supplementation on the broiler gut microbiota structure was dependent on the age of the chicken. At the same time, we observed that berberine levels present in the cecal lumen, which could potentially interact locally with the bacteria, were lower on day 21 than on day 12 posthatch. Accordingly, the antibiotic-like effects of berberine were less pronounced in older birds. Richness and diversity were reestablished in the cecum as no difference was observed between the groups and were even increased in the colon. Only some studies reported a certain degree of dysbiosis following berberine treatment in healthy animals or on intestinal bacteria *in vitro* (36, 61, 62). Other studies reported that berberine selectively enriched beneficial bacteria and along SCFAs production including butyrate, proposed by authors as contributing to the observed anti-inflammatory effects (11–13). Data variability related to which individual bacteria is affected by berberine might partly come from the different dosages used. In addition, in our study, we observed that an even higher dose of dietary berberine (2 g/kg feed) had a detrimental effect on the weight of birds. Further investigation is therefore needed to find an optimal dose of in-feed berberine that can control potential pathogens while promoting beneficial bacteria.

### Conclusion.

In summary, our results showed that dietary berberine at a high dose positively influenced the gut morphology of broilers as well as reduced intestinal inflammation. Berberine exerted an antibiotic-like effect by increasing the relative abundance of the family *Enterobacteriaceae* and decreasing the relative abundance of the family *Peptostreptococcaceae* as well as protective genera of the *Ruminococcaceae* and the *Lachnospiraceae* families, leading to a certain degree of dysbiosis, which did not reflect the observed beneficial effects on gut health. Further studies in broilers are imperative to determine whether a lower dose of berberine can induce beneficial host effects without causing a dysbiotic shift in the gut microbiota. The production of bioactive metabolites by the gut microbiota should also be investigated as part of berberine activity *in vivo*.

## MATERIALS AND METHODS

### Chemicals and reagents.

Berberine chloride and palmatine chloride (purity ≥98%) were obtained from Merck (Sigma-Aldrich, Overijse, Belgium). Berberine hydrochloride-d6 was obtained from Toronto Research Chemicals (North York, Canada). Stock solutions of all components at 1 mg/mL were prepared in methanol. In addition, a stock solution of berberine at 4 mg/mL was prepared in methanol, involved only in the quantification of berberine in cecal content. Stock solutions were stored at less than or equal to −15°C. Working solutions were prepared by dilution of these stock solutions in Milli-Q grade water and stored at 2 to 8°C. Solvents involved in sample extraction, acetonitrile, and methanol, were both of high-performance liquid chromatography grade (Fisher Scientific, Filter Service, Eupen, Belgium). Acetonitrile as the organic mobile phase component was of ULC/MS grade (Biosolve, Valkenswaard, the Netherlands). Formic acid, used in sample extraction as well as in mobile phase preparation, was also of ULC/MS grade (Biosolve). l-ascorbic acid (purity ≥98%) used in sample preparation was obtained from Merck (Sigma). Water of Milli-Q grade, used for preparation of the aqueous mobile phase component and dilution of chemicals, was produced in-house by a water purifying system Milli-Q-SP (Merck).

### Animals, experimental design, and dietary treatment.

The study was undertaken following the guidelines of the ethics committee of the Faculty of Veterinary Medicine and of Bioscience Engineering, Ghent University, in accordance with the EU Directive 2010/63/EU. One-day-old Ross 308 broilers were obtained from a local hatchery and housed in pens on wood shavings. They were allotted into three treatment groups (control group, BBR group, BBRHigh group), with three replicates (pens) per treatment group (4 chickens per replicate per sampling time point, 2 time points, so 8 chickens per pen at the beginning). The animals were not vaccinated. Water and commercial starter feed (day 1 to 12, FARM 1 Mash, Country’s Best, Versele Laga; Deinze, Belgium) or grower feed (day 13 to 21, FARM 2 mash, Country’s Best, Versele Laga; Deinze, Belgium) were provided *ad libitum*. The control group received the standard nonsupplemented diet, whereas the chickens in the BBR group and the BBRHigh group were fed the same feed supplemented with 1 g berberine/kg feed and 2 g berberine/kg feed, respectively, throughout the whole trial period. On day 12, 4 birds per pen were euthanized by sodium pentobarbital injection for sampling. Jejunal, ileal, cecal, and colonic contents were stored at −20°C for microbiota composition analysis (16S rRNA gene sequencing). Additionally, cecal content samples were collected and stored at −20°C for berberine and SCFA quantification. Duodenal and jejunal tissue samples were collected and fixed in 4% phosphate-buffered formaldehyde for histological analysis. Blood samples were collected in heparin tubes and centrifuged at 524 × *g* for 10 min to recover the plasma fraction to be stored at −20°C for berberine quantification. At day 21, the 4 remaining birds per pen were euthanized and the same samples were taken as at day 12. Birds were weighted at both time points (day 12: 275 ± 36 g control group, 284 ± 29 g BBR group, 177 ± 33 g BBRHigh group; day 21: 662 ± 78 g control group, 664 ± 101 g BBR group, 439 ± 88 g BBRHigh group). As the highest dose of berberine significantly decreased the weight of birds, this group was excluded from further analyses.

### DNA extraction from intestinal content.

DNA was extracted from intestinal content using the cetyltrimethylammonium bromide (CTAB) method as previously described with minor modifications (63). In brief, 200 mg of either jejunal or ileal content or 100 mg of cecal or colonic content was suspended in 500 μL CTAB buffer (hexade-cyltrimethylammonium bromide >98% (Sigma-Aldrich) 5% (wt/vol), 0.35 M NaCl, 120 nM K_2_HPO_4_) and 500 μL mL phenol-chloroform-isoamyl alcohol (25:24:1). The mixture was homogenized by grinding (2×) with 500 mg unwashed glass beads (Sigma-Aldrich) in a bead beater (2 min, 30 Hz for the ileal content, 1.5 min, 22.5 Hz for the other segments; TissueLyser; Qiagen, Hilden, Germany) with a 30-s interval between shakings. Samples were centrifuged for 10 min at 8,000 rpm, and 300 μL of the supernatant was transferred to a new tube. A second extraction from the remaining content was performed by adding 250 μL CTAB buffer and homogenizing and centrifuging the sample as described above. An equal volume (600 μL) of chloroform-isoamyl alcohol (24:1) was added to the supernatant collected to remove the phenol from the samples. The mixture was further centrifuged at 16,000 × *g* for 10 s. A 500-μL aliquot of the aqueous phase was transferred to a new tube. Nucleic acids were precipitated with 2 volumes of PEG-6000 solution (polyethylene glycol 30% wt/vol; 1.6 M NaCl) for 2 h at room temperature. Samples were centrifuged (13,000 × *g*, 20 min) and washed with 1 mL ice-cold ethanol (70% vol/vol). The pellet obtained was further centrifuged (13,000 × *g*, 20 min), dried, and resuspended in 100 μL deionized water for the ileal, cecal, and colonic DNA or 50 μL deionized water for the jejunal DNA (LiChrosolv Water; Merck, Darmstadt, Germany). The quality and the concentration of the DNA were examined spectrophotometrically (NanoDrop; Thermo Scientific, Waltham, MA, USA).

### 16S rRNA gene sequencing for microbiota composition analysis.

To characterize the taxonomic groups in the jejunal, ileal, cecal, and colonic microbiota of the chickens, the V3 to V4 hypervariable region of 16s rRNA gene was amplified using the gene-specific primers S-d-Bact-0341-b-S-17 (5′-TCGTCG GCA GCG TCA GAT GTG TAT AAG AGA CAG CCTACGGGNGGC WGC AG-3′) and S-d-Bact-0785-a-A-21 (5′-GTC TCG TGG GCT CGG AGA TGT GTA TAA GAGACA GGA CTACHVGGG TAT CTA ATC C-3′) (64). Each 25 μL of PCR contained 2.5 μL of DNA (~2 ng/μL), 0.2 μM each of the primers, and 12.5 μL 2 × KAPA HiFi HotStart ReadyMix (Kapa Biosystems, Wilmington, MA, USA). The program of PCR was set as follows: initial denaturation at 95°C for 3 min, followed by 25 cycles of 95°C for 30 s, 55°C for 30 s, 72°C for 30 s, and a final extension at 72°C for 5 min. The PCR products were purified using CleanNGS beads (CleanNA, Waddinxveen, The Netherlands). The DNA quantity and quality were analyzed spectrophotometrically (NanoDrop) and by agarose gel electrophoresis. A second PCR step was used to attach dual indices and Illumina sequencing adapters (i5 and i7 primers) to the 16S V3-V4 fragment in a 50-μL reaction volume containing 5 μL of purified PCR product, 25 μL of 2× KAPA HiFi HotStart ReadyMix, and 0.5 μM primers. The PCR conditions were the same as the first PCR with the number of cycles reduced to 8. The final PCR products were purified using the same method as above, and the concentration was determined using the Quantus double-stranded DNA assay (Promega, Madison, WI, USA). The final bar-coded libraries were combined into an equimolar 5-nM pool and sequenced using Illumina MiSeq v3 technology (2 × 300 bp, paired-end) at Macrogen (Gasan-dong, World Meridian I, Seoul, South Korea).

### Bioinformatics and statistical analysis of 16S rRNA gene amplicon data.

Demultiplexing of the amplicon data set and deletion of the barcodes was done by the sequencing provider. The quality of the raw sequence data was checked with the FastQC quality-control tool (Babraham Bioinformatics, Cambridge, UK) followed by initial quality filtering using Trimmomatic v0.38 by cutting reads with an average quality per base below 15 using a 4-base sliding window and discarding reads with a minimum length of 200 bp (65). The paired-end sequences were assembled, and primers were removed using PANDAseq (66), with a quality threshold of 0.9 and length cutoff values for the merged sequences between 390 and 430 bp. Chimeric sequences were removed using UCHIME (67). Open-reference OTU picking was performed at 97% sequence similarity using USEARCH (v6.1) and converted to an OTU table (68). OTU taxonomy was assigned against the Silva database (v128, clustered at 97% identity) (69) using the PyNast algorithm with QIIME (v1.9.1) default parameters (70). OTUs with a total abundance below 0.01% of the total sequences were discarded (71), resulting in a mean average of 34430.243 reads per sample. Alpha rarefaction curves were generated using the QIIME “alpha_rarefaction.py” script, and a subsampling depth of 3,500 reads was selected. After excluding 3 missing samples and 10 samples with insufficient sequencing depth, 179 samples were left for the analysis. On day 12, there were 11, 12, 12, and 11 samples in the control group and 11, 12, 12, and 12 samples in the BBR group for jejunum, ileum, cecum, and colon, respectively. On day 21, there were 11, 12, 10, and 11 samples in the control group and 11, 12, 11, and 11 samples in the BBR group for jejunum, ileum, cecum, and colon, respectively.

All further analyses of microbiomes included “Pen” as a covariate or random factor, as appropriate. Analyses of alpha diversity (Observed OTUs, Chao1 richness estimator, and Shannon diversity estimator) and beta diversity (Bray-Curtis, unweighted UniFrac dissimilarities) were performed using the *phyloseq* (72) (v1.30.0) pipeline in R (v3.6.1). A linear mixed model was used to compare alpha diversity data, using the *lmer()* function of the *lme4* package (v1.1-26). Differences in beta diversity were examined by PERMANOVA using the *adonis* function from the *vegan* package (v2.5-7). To detect differentially abundant taxa between the different diet groups, LinDA (v0.1.0) was applied to the centered log-ratio transformed, nonrarefied, community composition data for all intestinal segment communities (73). For all tests, a *P* value of <0.05 was considered significant.

### Metabolic function prediction of the microbial communities.

To gain more insight into the effect of berberine on the possible functional pathways of the microbial communities, the functional composition was predicted using PICRUSt (Phylogenetic Investigation of Communities by Reconstruction of Unobserved States) (74). PICRUSt uses precomputed ancestral state reconstructions based on the Greengenes database. Therefore, OTU picking was reperformed as described above with the following modifications: closed-reference OTU picking was used, and OTU taxonomy was assigned against the Greengenes database (v 13.5) (75) after which the OTU counts were normalized by their expected 16S copy number using QIIME (76, 77). Metagenome predictions were performed against the KEGG database (78). The resulting KOs were further summarized into functional modules based on the GMM database using GoMixer (Raes Lab). Differentially represented GMMs were detected using LinDA as above. The contribution of bacterial families to different GMMs was computed with the script “metagenome_contributions.py.” To investigate the nitroreductase activity, the UniProt database was screened for nitroreductases and their associated KOs. Five KOs (K00118, K07078, K09019, K10678, and K10679) were present in our data set and further analyzed.

### SCFA quantification.

The amount of acetate, propionate, butyrate, valerate, isobutyrate, and isovalerate was quantified using gas chromatography as previously described (79). In short, SCFAs were extracted from cecal content using diethyl ether, after the addition of 2-methyl hexanoic acid as an internal standard. Extracts were analyzed using a GC-2014 gas chromatograph (Shimadzu, ‘s-Hertogenbosch, the Netherlands), equipped with a capillary fatty-acid free EC-1000 Econo-Cap column (Alltech, Laarne, Belgium), a flame ionization detector and a split injector.

### Determination of berberine in plasma and cecal samples.

**(i) Instrument and experimental conditions.** The UPLC instrument consisted of an Acquity H-Class Quaternary Solvent Manager and an Acquity FTN Sample Manager, purchased from Waters (Berchem, Belgium). For chromatographic separation, an Acquity UPLC HSS T3 column (1.8 μm, 100 mm × 2.1 inner diameter [ID]) was used, protected by a precolumn of the same type (VanGuard, 5 mm × 2.1 mm ID), both obtained from Waters. A gradient elution was performed with a mobile phase of 0.1% (vol/vol) formic acid in water (A) and acetonitrile (B) at a flow rate of 0.3 mL/min. The gradient program used was as follows: 0 min: 80% A, 20% B; 0 to 5.0 min: 80% A, 20% B to 55% A, 45% B; 5.0 to 5.1 min: 55% A, 45% B to 10% A, 90% B; 5.1 to 10.9 min: 10% A, 90% B; 10.9 to 11.0 min: from 10% A, 90% B to 80% A, 20% B; and 11.0 to 15.0 min: 80% A, 20% B. The column temperature was maintained at 25°C. The autosampler temperature was set at 10°C. The UPLC effluent was sent from 2.5 min to 5.5 min, by use of a divert valve, to a Xevo TQ-S triple quadrupole mass spectrometer (Waters), equipped with an electrospray ionization ion source. The UPLC-MS/MS analysis was run under the control of MassLynx software (version 4.1), which was also used for subsequent data processing. Operating conditions for the ESI source used in the positive ionization mode were optimized by direct infusion of all individual components in combination with the mobile phase at 50% A/50% B. The following tune parameters were used for the detection of all components: capillary voltage, 3 kV; cone voltage, 10 V; source offset, 50 V; source temperature, 150°C; desolvation temperature, 500°C; desolvation gas flow, 800 L/h; cone gas flow, 150 L/h; collision gas flow, 0.2 mL/min; ion energy 1 and 2, 0.5; LM 1 and LM 2 resolution, 3; and HM 1 and HM 2 resolution, 15. Components were detected in MS/MS mode using component-specific multiple reaction monitoring (MRM) transitions: for berberine at *m/z* 336.14 > 320.16 (quan-ion for quantification purposes) and 336.14 > 292.12 (qual-ion, for identification purposes), for berberine-d6 at *m/z* 342.16 > 294.19, and for palmatine at *m/z* 352.19 > 336.16, all recorded at a 30-eV collision energy. Quantification of berberine was performed using the response, i.e., the ratio of the peak area of berberine to the peak area of the internal standard, which was berberine-d6 for plasma, and palmatine for cecal contents.

**(ii) Preparation of plasma samples.** A 137.5-μL sample was used for sample extraction, which consisted of 125 μL of plasma to which, after *in vivo* sampling, 12.5 μL of ascorbic acid 170 mg/mL solution in water was added, before storage at less than or equal to −15°C until UPLC-MS/MS analysis. The ascorbic acid solution was added to prevent oxidation of dihydroberberine to berberine during sample storage (according to reference 15). The plasma sample was transferred to an Ostro 96 well-plate (25 mg) (Waters). Subsequently, 25 μL of the internal standard solution containing 100 ng/mL of berberine-d6 was added. A 490-μL volume of 1% (vol/vol) formic acid solution in acetonitrile (1/3 ratio) was added, followed by gentle mixing. Vacuum was applied on the Ostro plate for 5 min, and the eluate was collected in a 2 mL square 96-well collector plate. The sample was transferred in a glass tube and evaporated at 40°C under a gentle stream of nitrogen in a Pierce (Rockford, IL, USA) Reacti-Therm III Heating Module and Reacti-Vap III Module. The dried sample extract was reconstituted in a 450-μL volume of Milli-Q water supplemented with 50 μL ascorbic acid 170 mg/mL solution, followed by injection of a 5-μL sample aliquot onto the UPLC-MS/MS apparatus. In berberine-supplemented animals, some samples showed berberine levels above the upper calibration limit of 100 ng/mL. For these samples, 25 μL of an internal standard solution containing 1 μg/mL of berberine-d6 was added (10 times the normal amount), while the remaining part of the sample extraction procedure remained unchanged. The final extract was redissolved in a 10 times higher volume, 4,500 μL of Milli-Q water supplemented with 500 μL ascorbic acid 170 mg/mL solution.

**(iii) Calibration and quality control preparation for determination of berberine in plasma.** Calibration standards and quality control (QC) samples were prepared as follows: 125 μL of blank plasma were supplemented with 12.5 μL of ascorbic acid 170 mg/mL solution and 25 μL of berberine working solution at different levels (5, 25, 50, 125, 250, and 500 ng/mL). The calibration curve was in the 1- to 100-ng/mL range, including concentrations of 1, 5, 10, 25, 25, and 100 ng/mL. The concentrations of QC for berberine were 1 ng/mL (low), 10 ng/mL (medium), and 100 ng/mL (high). The applicability of the dilution procedure for highly concentrated plasma samples mentioned in the previous section was verified by the preparation of QC samples at 1,000 ng/mL of berberine.

**(iv) Preparation of cecal content samples.** Cecum content (250 mg) was weighed in a 15-mL polypropylene centrifuge tube and supplemented with 250 μL of ascorbic acid 170 mg/mL solution for stabilization of dihydroberberine, followed by brief vortexing. Subsequently, 125 μL of an internal standard solution containing 2 μg/mL of palmatine in water was added, followed by brief vortexing. A 2.5-mL volume of extraction solvent was then added, consisting of a 1% (vol/vol) formic acid solution in methanol. The sample was put on a rotary shaker set at 80 rpm for 20 min, followed by centrifugation (4,000 rpm, 10 min, 4°C). The supernatant was then diluted by a factor 20 by transferring 50 μL of supernatant in an autosampler vial, followed by the addition of 100 μL of ascorbic acid 170 mg/mL solution and 850 μL of Milli-Q water and brief vortex mixing. A 5-μL aliquot of this diluted sample extract was injected into the UPLC-MS/MS instrument. Samples from berberine-supplemented animals presented berberine levels higher than the calibration limit of 5,000 ng/g. For these samples, 125 μL of an internal standard solution containing 200 μg/mL of palmatine was added (100 times the normal amount), while the remaining part of the sample extraction procedure remained unchanged. The supernatant was then diluted by a factor 2,000, by diluting first 250 μL of sample extract in a 25-mL final volume with Milli-Q water. Further dilution was achieved by transferring 50 μL of the above solution in an autosampler vial, followed by the addition of 100 μL of ascorbic acid 170 mg/mL solution and 850 μL of Milli-Q water and brief vortex mixing.

**(v) Calibration and QC preparation for determination of berberine in cecal content.** Calibration standards and QC samples were prepared as follows: 250 mg blank cecal content was supplemented with 250 μL of ascorbic acid 170 mg/mL solution and 125 μL berberine working solution at different levels (0.2, 0.5, 1, 2, 5, and 10 μg/mL). The calibration curve was in the 100- to 5,000-ng/g range, including concentrations of 100, 250, 500, 1,000, 2,500, and 5,000 ng/g. The concentrations of QC for berberine were 100 ng/g (low), 500 ng/g (medium), and 5,000 ng/g (high). The applicability of the dilution procedure for highly concentrated cecal samples was verified by the preparation of QC samples at 500 μg/g.

**(vi) Method validation.** The UPLC-MS/MS analysis method for the quantification of berberine both in plasma and intestinal contents was validated according to the guidelines of the European Medicines Agency, i.e., of the Committee for Medicinal Products for Veterinary Use (EMEA/CVMP/VICH/463202/2009) and of the Committee for Medicinal Products for Human Use (EMEA/CHMP/EWP/192217/20), in accordance with the EU Directive 2002/657/EC. The following parameters were evaluated: linearity, within-day and between-day precision and accuracy, limit of quantification (LOQ), limit of detection (LOD), dilution integrity, extraction recovery, matrix effect, carryover, specificity, and storage stability parameters. The results of the method validation can be found in Supplemental Methods S1. It is shown that a reliable and reproducible method for detecting berberine in plasma and intestinal content was developed. LOQ and LOD of berberine were 0.1 ng/mL and 0.02 ng/mL for plasma and 100 ng/g and 2.7 ng/g for cecal content.

### Intestinal morphology.

Twenty-four-hour formalin-fixed duodenal and jejunal intestinal tissue segments were embedded in paraffin wax and sectioned at 5 μm, followed by hematoxylin and eosin staining. Villus length and crypt depth were assessed using a PC-based image analysis system (Leica Application Suite V4, LAS V4; Leica, Diegem, Belgium). Measurements were performed on at least 9 randomly selected villi/crypt apparently not exhibiting mechanical damage, when present, after which the average per animal was calculated.

### CD3 immunohistochemistry.

Slides for immunohistochemical staining for CD3^+^ T-cells were automatically deparaffinized (Shandon Varistain-Gemini) before antigen retrieval with a pressure cooker in citrate buffer (10 mM, pH 6). Endogenous peroxidase activity was blocked by treating the slides with peroxidase blocking reagent (S2023; Dako, Glostrup, Denmark) for 5 min. The presence of T-cells (CD3-positive cell abundance) in duodenal tissue was evaluated using polyclonal primary antibodies against CD3 (A0452; Dako; 1:100 dilution, 30 min at room temperature), followed by incubation with a secondary labeled polymer-horseradish peroxidase (HRP) anti-rabbit (Envision + System-HRP [DAB; K4011], 30 min at room temperature). Slides were evaluated using the computer-based image analysis program LAS V4.1. The CD3^+^ area percentage in the duodenal tissue was quantified using four representative fields of view per intestinal section (×10 objective).

### *In vitro* berberine susceptibility testing.

Based on the 16S sequencing data, strains from 9 different genera were selected for a berberine susceptibility assay. Strains were chosen among bacterial genera that were significantly differentially abundant in the cecum or the colon, either enhanced (Escherichia, Klebsiella, Proteus) or decreased (*Faecalibacterium*, *Anaerostipes*, *Subdoligranulum*, *Eubacterium*) by berberine treatment. This included three intestinal facultative anaerobes, Escherichia coli (ATCC 25922), Klebsiella pneumoniae (dog isolate), and Proteus mirabilis (chicken isolate), and four intestinal strict anaerobes, Anaerostipes butyraticus (chicken isolate 35-7e; reference 80), *Faecalibacteriuum prausnitzii* (A2-165), Subdoligranulum variabile (chicken isolate 40-4c; reference 80), and Eubacterium hallii (chicken isolate 33-7; reference 80). The anaerobe Clostridium perfringens (EHE-NE18; reference 81) was also tested as berberine was previously shown to alleviate C. perfringens-induced necrotic enteritis *in vivo* (26). The animal isolates are derived from the diagnostic bacteriology of the Laboratory of Veterinary Bacteriology, Ghent University. Before testing, the strains were subcultured overnight in M2GSC medium pH 6 containing per 100 mL, 15 mL of rumen fluid, 1 g of casitone, 0.25 g of yeast extract, 0.2 g of glucose, 0.2 g of cellobiose, 0.2 g of soluble starch, 0.045 g of K_2_HPO_4_, 0.045 g of KH_2_PO_4_, 0.09 g of (NH_4_)2SO_4_, 0.09 g of NaCl, 0.009 g of MgSO_4_·7H_2_O, 0.009 g of CaCl^2^, 0.1 mg of resazurin, 0.4 g of NaHCO_3_, and 0.1 g of cysteine hydrochloride (82). Inocula were then 1:1,000 diluted and exposed to 2-fold serial dilution of berberine concentrations ranging from 225.0 to 7.1 μg/mL using the broth microdilution method. Cultures were incubated for 24 h, then absorbance was measured at 600 nm to assess bacterial growth. All experiments were performed in triplicate at 37 to 40°C under anaerobic conditions to mimic the physiological conditions of the broiler cecum.

### Statistical analysis.

Statistical analysis of the gut microbiota was performed using R, as described above. The chicken phenotypes were also analyzed with R with linear mixed models, with “Pen” as a random factor, using the *lmer()* function of the *lme4* package (v1.1-26). Spearman correlation coefficients were studied with the *cor.test()* and the *ggscatter()* function from the *ggpubr* R package (v0.4.0) to analyze the relationship between parameters. Additionally, *in vitro* bacterial growth data were analyzed using GraphPad Prism software (version 8.4.3, San Diego CA, California), with one-way ANOVA, followed by Dunnett’s multiple-comparison test where appropriate, to compare growth in the presence of various concentrations berberine to the control. For all tests, a *P* value of <0.05 was considered significant.

### Data availability.

The raw sequencing data and corresponding metadata are available on NCBI SRA under the BioProject PRJNA905932.
