# Supplementary material for: A High Dose of Dietary Berberine Improves Gut Wall Morphology, Despite an Expansion of Enterobacteriaceae and a Reduction in Beneficial Microbiota in Broiler Chickens
Source: mSystems. 2023 Jan 31;8(1):e01239-22. doi: 10.1128/msystems.01239-22 (PMC9948737; doi:10.1128/msystems.01239-22)
Supplement: TEXT S1 [file msystems.01239-22-s0001.docx]

**Supplemental Methods S1**

- **Linearity of berberine in chicken plasma and caecal content (n =3)**

| Matrix | Calibration  range* | Evaluation occasion | a | b | r | GoF (%) |
| --- | --- | --- | --- | --- | --- | --- |
| Plasma | 1-100 | Day 1 | 0.10041 | -0.01437 | 1.000 | 4.4 |
|  |  | Day 2 | 0.09830 | -0.01965 | 1.000 | 3.0 |
|  |  | Day 3 | 0.09569 | -0.01039 | 1.000 | 2.7 |
| Caecum | 100-5000 | Day 1 | 0.001373 | -0.01149 | 0.998 | 4.4 |
|  |  | Day 2 | 0.001417 | -0.01741 | 0.999 | 3.8 |
|  |  | Day 3 | 0.001401 | -0.01580 | 0.999 | 3.7 |

*ng/ml for plasma, ng/g for caecal content. Matrix-matched calibration curves were prepared on three different analysis days. Calibration curves were best described with a linear calibration model y= ax + b, using a 1/x fit weighting for plasma, and a 1/x^2^ fit weighting for caecal content. r represents the correlation coefficient and GoF the goodness-of-fit. r was above the 0.99 criterion, while the GoF was below the 20 % (for plasma) or 10 % (for caecal content) criterion.

- **Within-day (n = 6) and between-day precision and accuracy of berberine in chicken plasma and caecal content**

| Matrix | Theoretical  concentration* | Evaluation occasion | Mean concentration* | SD | RSD  (%) | Accuracy (%) |
| --- | --- | --- | --- | --- | --- | --- |
| Plasma | 1 | Within-run, day 1 | 1.00 | 0.033 | 3.3 | + 0.2 |
|  |  | Within-run, day 2 | 1.04 | 0.022 | 2.1 | + 4.3 |
|  |  | Within-run, day 3 | 1.03 | 0.060 | 5.8 | + 3.2 |
|  |  | Between-run | 1.03 | 0.043 | 4.2 | + 2.6 |
|  | 10 | Within-run, day 1 | 9.4 | 0.17 | 1.8 | - 5.5 |
|  |  | Within-run, day 2 | 9.5 | 0.15 | 1.6 | - 4.9 |
|  |  | Within-run, day 3 | 9.7 | 0.30 | 3.1 | - 3.3 |
|  |  | Between-run | 9.5 | 0.22 | 2.4 | - 4.6 |
|  | 100 | Within-run, day 1 | 101.3 | 2.4 | 2.3 | + 1.3 |
|  |  | Within-run, day 2 | 99.3 | 0.7 | 0.7 | - 0.7 |
|  |  | Within-run, day 3 | 99.3 | 1.1 | 1.1 | - 0.7 |
|  |  | Between-run | 100.0 | 1.7 | 1.7 | 0.0 |
| Caecum | 100 | Within-run, day 1 | 103.9 | 1.1 | 1.1 | + 3.9 |
|  |  | Within-run, day 2 | 105.6 | 2.3 | 2.2 | + 5.6 |
|  |  | Within-run, day 3 | 104.0 | 2.3 | 2.2 | + 4.0 |
|  |  | Between-run | 104.5 | 2.0 | 2.0 | + 4.5 |
|  | 500 | Within-run, day 1 | 514.2 | 3.4 | 0.7 | + 2.8 |
|  |  | Within-run, day 2 | 494.4 | 3.1 | 0.6 | - 1.1 |
|  |  | Within-run, day 3 | 490.5 | 9.5 | 1.9 | - 1.9 |
|  |  | Between-run | 499.7 | 12.1 | 2.4 | - 0.1 |
|  | 5000 | Within-run, day 1 | 5201.9 | 28.6 | 0.6 | + 4.0 |
|  |  | Within-run, day 2 | 5040.2 | 72.0 | 1.4 | + 0.8 |
|  |  | Within-run, day 3 | 5139.0 | 91.8 | 1.8 | + 2.8 |
|  |  | Between-run | 5127.0 | 94.5 | 1.8 | + 2.5 |

*ng/ml for plasma, ng/g for caecal content. SD: standard deviation, RSD: relative standard deviation. Criteria for precision and accuracy were met at all levels tested, in both matrices.

- **Limit of Quantification (LOQ) and Limit of Detection (LOD)**

The LOQ was the smallest measured concentration of berberine above which the accuracy and precision were acceptable. LOQ of berberine was fixed at 100 ng/g in caecal contents, based on above results. LOQ of berberine in plasma was further reduced at 0.1 ng/mL.

The LOD was the smallest measured concentration of berberine from which it was possible to deduce the presence with acceptable certainty. It was determined by calculating the theoretical analyte concentration that corresponded with a signal-to-noise (S/N) ratio of 3/1, based on the S/N ratio of berberine in the LOQ samples. The LOD of berberine was 0.02 ng/ml in plasma, and 2.7 ng/g in caecal contents.

- **Within-day (n = 6) and between-day precision and accuracy of berberine in chicken plasma at the tested 0.1 ng/ml LOQ level**

| Matrix | Theoretical  concentration* | Evaluation occasion | Mean concentration* | SD | RSD  (%) | Accuracy (%) |
| --- | --- | --- | --- | --- | --- | --- |
| Plasma | 0.1 | Within-run, day 1 | 0.103 | 0.013 | 12.2 | + 3.2 |
|  |  | Within-run, day 2 | 0.095 | 0.013 | 13.4 | - 4.7 |
|  |  | Within-run, day 3 | 0.119 | 0.013 | 10.6 | + 19.0 |
|  |  | Between-run | 0.106 | 0.016 | 14.9 | + 6.0 |

*ng/ml. Criteria for precision (within-run <30%, between-run < 45%) and accuracy (– 50 % to + 20 %) at this level were met.

- **Carry-over and specificity**

Carry-over was evaluated by the injection of a solvent sample after the highest calibrator sample. Specificity of the method was evaluated by analysing a blank matrix sample extract. For both parameters, and for both matrices, no peaks were detected at the retention times of berberine, palmatine, or berberine-d6.

- **Dilution integrity in chicken plasma and caecal content (n=6)**

| Matrix | Theoretical  concentration* | Mean concentration* | SD | RSD  (%) | Accuracy (%) |
| --- | --- | --- | --- | --- | --- |
| Plasma | 1000 | 1085.9 | 11.8 | 1.1 | + 8.6 |
| Caecum | 500 | 477.6 | 4.0 | 0.8 | - 4.5 |

*ng/ml for plasma, µg/g for caecal content. Criteria for precision (< 10 %) and accuracy (– 20 % to + 10 %) were met in both matrices. All caecum intestinal content samples of berberine-treated animals were analysed in this way, as well as a limited number of plasma samples above the highest 100 ng/ml calibrator sample.

- **Freeze/thaw (3 cycles) and bench top (room temperature, T = 4h) stability (n=6)**

| Matrix | Theoretical  concentration* | Stability  parameter | Mean concentration* | SD | RSD  (%) | Accuracy (%) |
| --- | --- | --- | --- | --- | --- | --- |
| Plasma | 10 | Freeze/thaw | 9.2 | 0.24 | 2.6 | - 8.3 |
|  | 10 | Bench top | 10.0 | 0.20 | 2.0 | + 0.1 |
| Caecum | 500 | Freeze/thaw | 483.6 | 9.9 | 2.1 | - 3.3 |
|  | 500 | Bench top | 434.7 | 3.2 | 0.7 | - 13.1 |

*ng/ml for plasma, ng/g for caecal content. Freeze/thaw stability during three cycles (≤ -15 °C to room temperature), and short-term stability in matrix at room temperature for 4 h (bench top stability) were evaluated, at the medium QC level, 10 ng/ml for plasma, and 500 ng/g for caecal contents. Criteria for precision (< 15 %, < 10 % respectively) and accuracy (- 30 % to + 10 %, – 20 % to + 10 % respectively) were met.

- **Stability of berberine in processed plasma or content extracts, stored at 10 °C in autosampler for 72 and 96 h respectively (n=3)**

| Matrix | Theoretical  concentation | Mean concentration | SD | RSD  (%) | Accuracy (%) |
| --- | --- | --- | --- | --- | --- |
| Plasma | 1 | 0.85 | 0.035 | 4.1 | - 15.0 |
|  | 10 | 9.6 | 0.21 | 2.1 | - 4.2 |
|  | 100 | 103.5 | 3.1 | 3.0 | + 3.5 |
|  | 1000 | 1095.3 | 3.9 | 0.4 | + 9.5 |
| Caecum | 100 | 102.5 | 1.5 | 1.4 | + 2.5 |
|  | 500 | 505.0 | 1.4 | 0.3 | + 1.0 |
|  | 5000 | 5146.4 | 58.9 | 1.1 | + 2.9 |
|  | 500000 | 496850 | 3144.5 | 0.6 | - 0.6 |

* ng/ml for plasma, ng/g for caecum intestinal contents. Stability in extract was evaluated at low, medium, and high QC levels after storage of the processed sample extract for 3 days (plasma) or 4 days (caecal content) in the autosampler at 10 °C. Criteria for precision and accuracy were met.

- **Extraction recovery (RE) and matrix effect (ME)**

RE and ME were evaluated based on the post-extraction spike method (82, 83), evaluation was done on the medium QC level of 10 ng/ml for plasma, and 500 ng/g for caecal content (n=6). Recovery was defined as the berberine response in a blank sample spiked and extracted subsequently, compared to the response in a blank sample extracted and spiked post-extraction at the same concentration. The matrix effect was obtained by comparing the berberine response in a blank sample extracted and spiked post-extraction to the response of a standard solution containing the same concentration and having the same solvent composition. In plasma, berberine recovery was found to be 82.9%, and matrix effect 93.5%. Comparable results were obtained for the related internal standard berberine-d6, 82.8% for RE and 95.6% for ME. In caecal contents, berberine recovery was 92.7%, and matrix effect 136.5%. Comparable results were obtained for the related internal standard, palmatine, 96.8% for RE and 144.7% for ME. These results indicate a good recovery of berberine from plasma and caecal content matrices, a limited signal suppression of both berberine and the related IS in plasma matrix, and a significant signal enhancement on the other hand in caecal content matrix, observed for both berberine and the related IS palmatine.
