# Supplementary material for: A High Dose of Dietary Berberine Improves Gut Wall Morphology, Despite an Expansion of Enterobacteriaceae and a Reduction in Beneficial Microbiota in Broiler Chickens
Source: mSystems. 2023 Jan 31;8(1):e01239-22. doi: 10.1128/msystems.01239-22 (PMC9948737; doi:10.1128/msystems.01239-22)
Supplement: TABLE S1 [file msystems.01239-22-s0002.docx]

**Table S1**

| **Microbiota** | **Bray-Curtis** | | **unweighted UniFrac** | |
| --- | --- | --- | --- | --- |
|  | **R^2^** | **p-value** | **R^2^** | **p-value** |
| **Jejunum** |  |  |  |  |
| Supplementation | 7.9% | 0.003 | 3.8% | 0.021 |
| Age | 13.9% | 0.001 | 11.5% | 0.001 |
| Interaction | 3.9% | 0.065 | 4.8% | **0.005** |
| Residual | 74.3% |  | 79.9% |  |
| **Ileum** |  |  |  |  |
| Supplementation | 8.0% | 0.001 | 6.2% | 0.001 |
| Age | 12.2% | 0.001 | 14.4% | 0.001 |
| Interaction | 7.3% | **0.006** | 4.7% | **0.002** |
| Residual | 72.5% |  | 74.7% |  |
| **Caecum** |  |  |  |  |
| Supplementation | 12.8% | 0.001 | 10.5% | 0.001 |
| Age | 17.2% | 0.001 | 17.7% | 0.001 |
| Interaction | 6.6% | **0.001** | 7.1% | **0.002** |
| Residual | 63.3% |  | 64.8% |  |
| **Colon** |  |  |  |  |
| Supplementation | 16.8% | 0.001 | 8.7% | 0.001 |
| Age | 6.0% | 0.009 | 12.1% | 0.001 |
| Interaction | 5.7% | **0.01** | 6.9% | **0.001** |
| Residual | 71.6% |  | 72.3% |  |

p-values were calculated on 999 possible permutations. R^2^ represents the percentage of variation accountable for the supplementation of berberine in the feed, the age or the interaction term.
