# Supplementary figures and images for: A High Dose of Dietary Berberine Improves Gut Wall Morphology, Despite an Expansion of Enterobacteriaceae and a Reduction in Beneficial Microbiota in Broiler Chickens
Source: mSystems. 2023 Jan 31;8(1):e01239-22. doi: 10.1128/msystems.01239-22 (PMC9948737; doi:10.1128/msystems.01239-22)

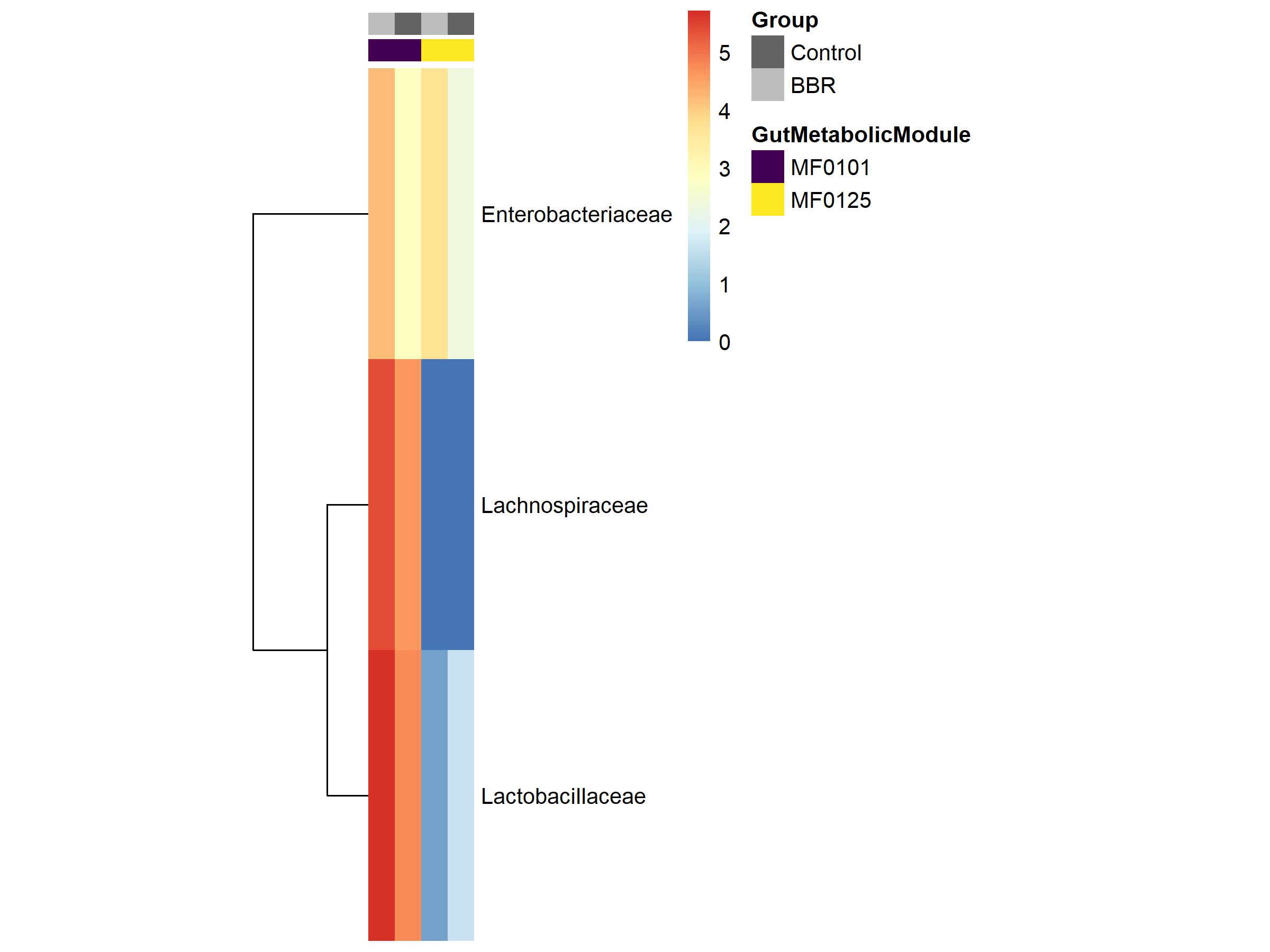

Supplement: FIG S1 [file msystems.01239-22-s0003.tif]

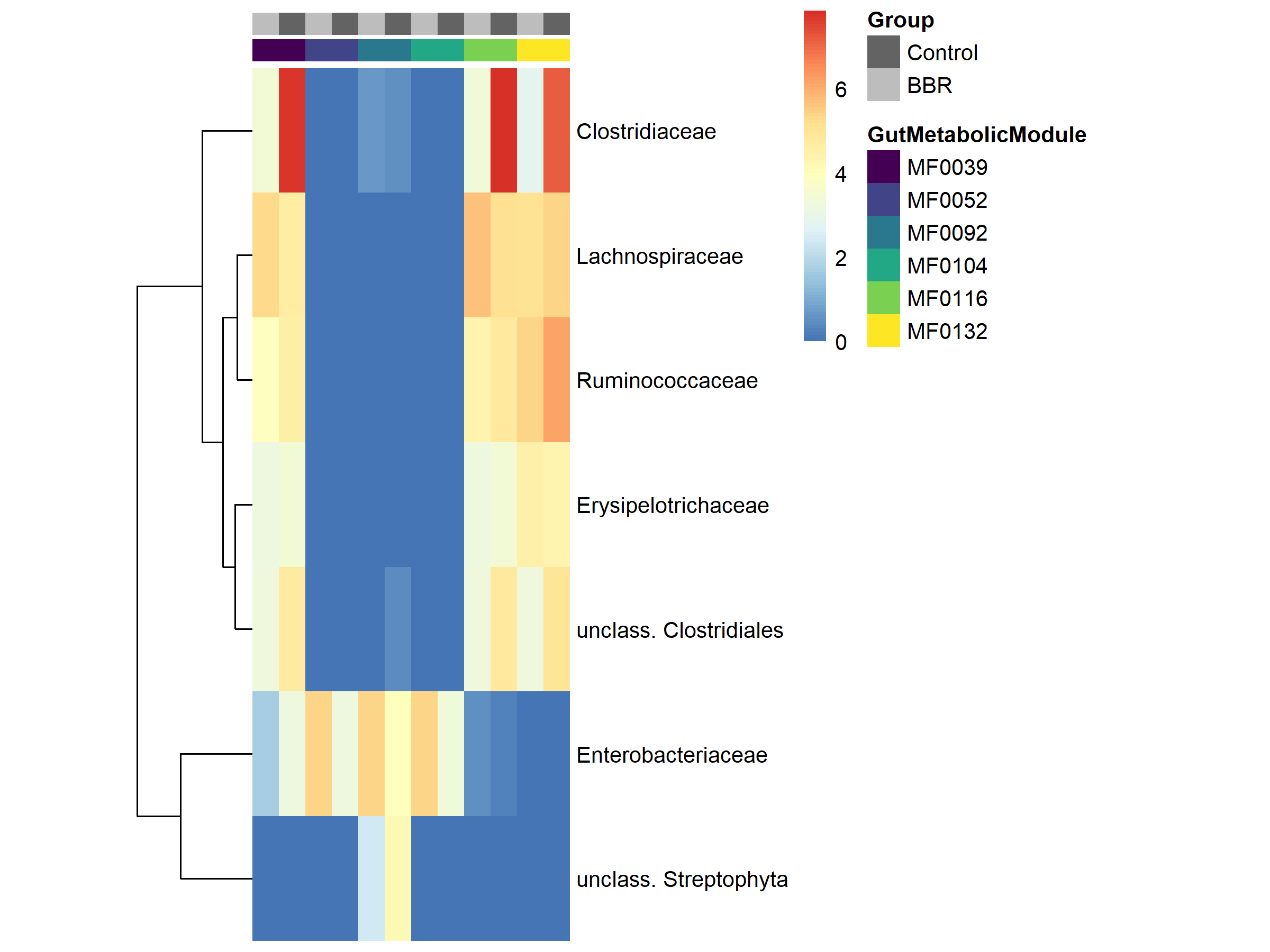

Supplement: FIG S2 [file msystems.01239-22-s0004.tif]

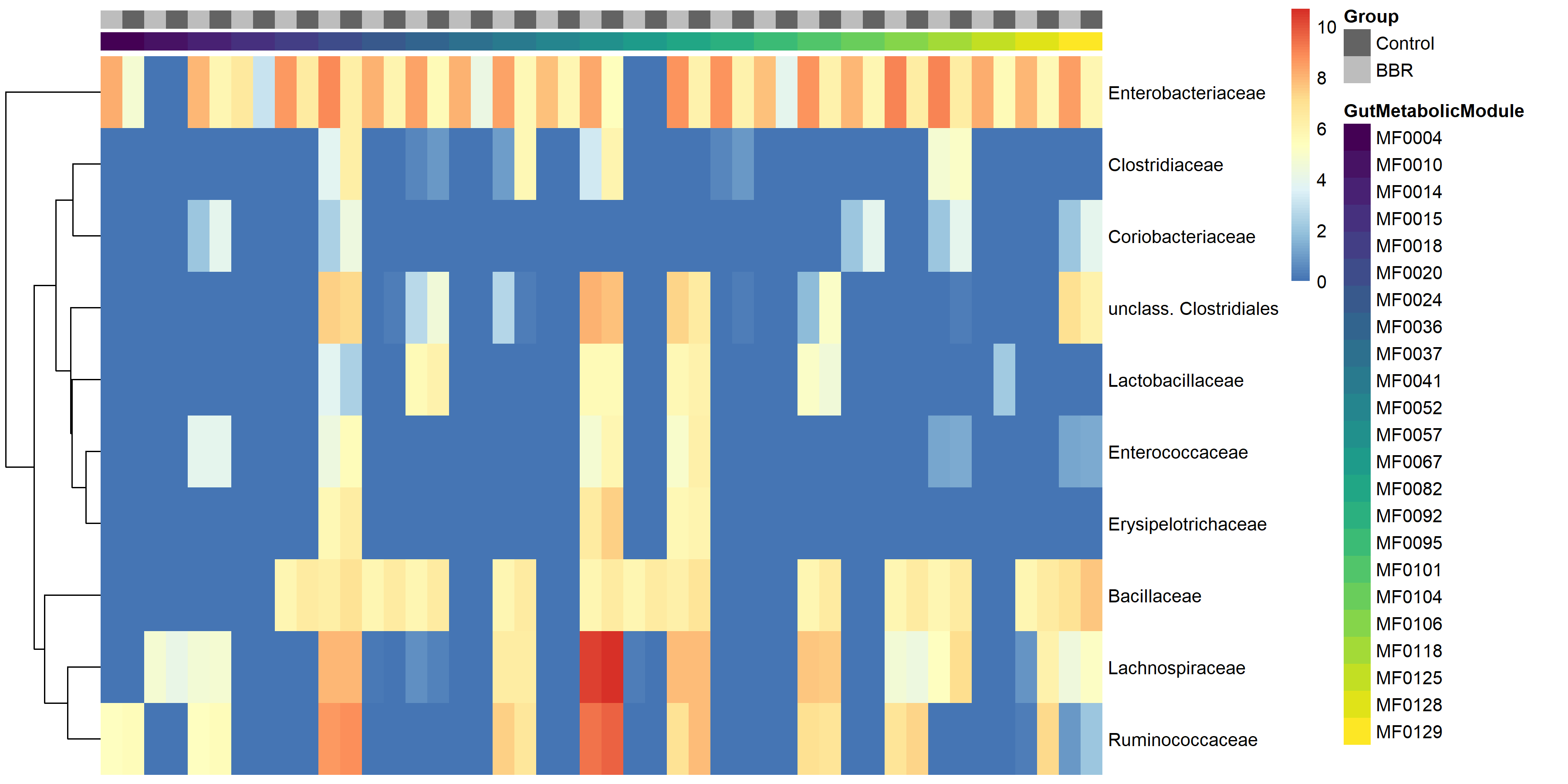

Supplement: FIG S3 [file msystems.01239-22-s0005.tif]

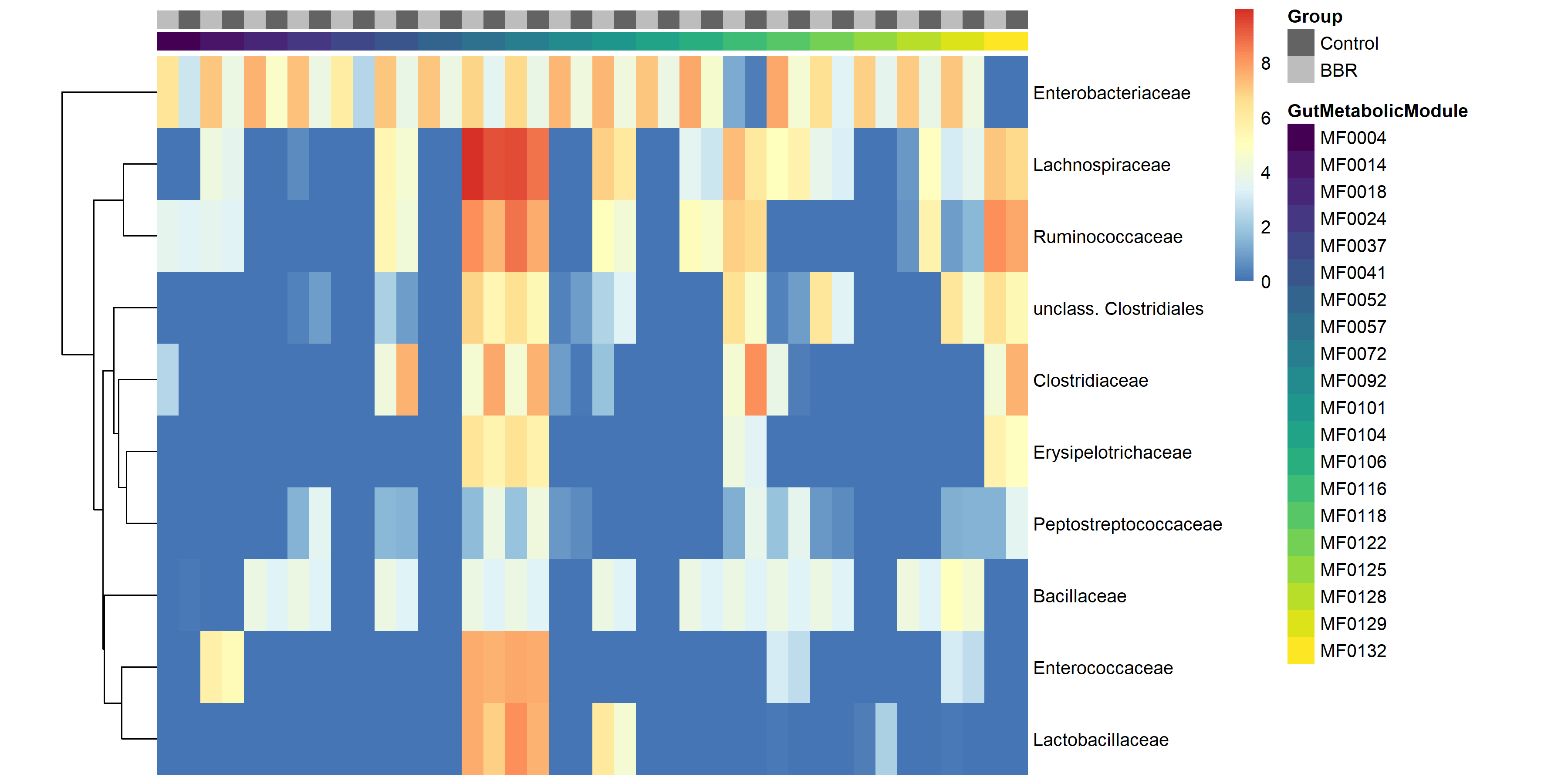

Supplement: FIG S4 [file msystems.01239-22-s0006.tif]

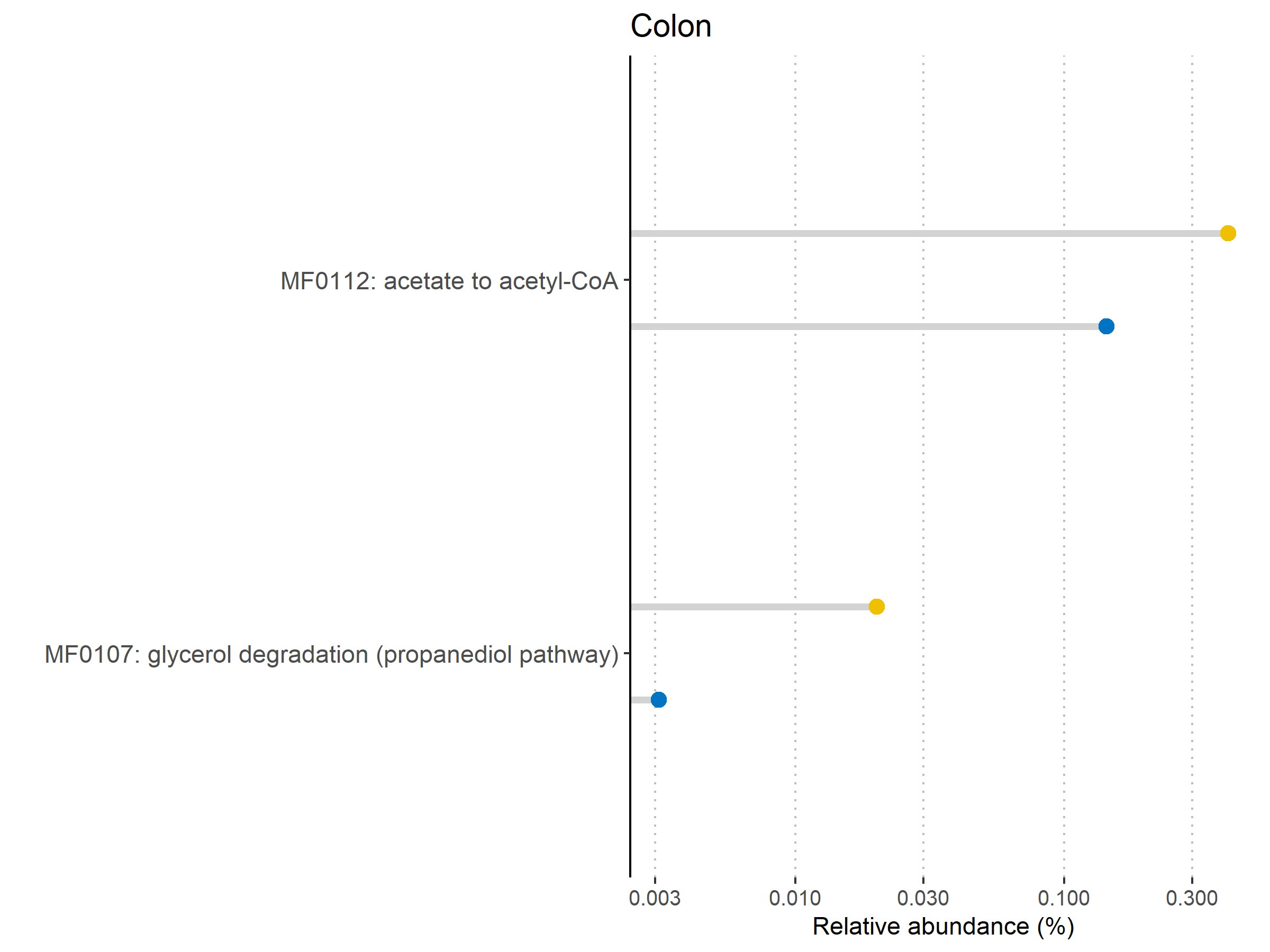

Supplement: FIG S5 [file msystems.01239-22-s0007.tif]
